# Effects of Mexican *Ganoderma lucidum* extracts on liver, kidney, and the gut microbiota of Wistar rats: A repeated dose oral toxicity study

**DOI:** 10.1371/journal.pone.0283605

**Published:** 2023-04-06

**Authors:** María E. Meneses, Daniel Martínez-Carrera, Laura González-Ibáñez, Nimbe Torres, Mónica Sánchez-Tapia, Claudia C. Márquez-Mota, Gilmar Rendón, Vladimir Mitzi, Alfredo Morales, Isaac Tello-Salgado, Armando R. Tovar

**Affiliations:** 1 CONACYT – Colegio de Postgraduados, *Campus* Puebla, Puebla, Mexico; 2 Centro de Biotecnología de Hongos Comestibles, Funcionales y Medicinales (CB-HCFM), *Campus* Puebla, Colegio de Postgraduados (CP), Puebla, Mexico; 3 Departamento de Fisiología de la Nutrición, Instituto Nacional de Ciencias Médicas y Nutrición Salvador Zubirán (INCMNSZ), Mexico City, Mexico; 4 Departamento de Nutrición Animal y Bioquímica, FMVZ, UNAM, Mexico City, Mexico; 5 Micología, CIB, Universidad Autónoma del Estado de Morelos, Morelos, Mexico; Institute of medical research and medicinal plant studies, CAMEROON

## Abstract

Well-characterized and standardized extracts of a Mexican genotype of *Ganoderma lucidum* (*Gl*), a medicinal mushroom, cultivated on oak sawdust (*Gl*-1) or oak sawdust plus acetylsalicylic acid (*Gl*-2, ASA), have been shown to exert antioxidant, hypocholesterolemic, anti-inflammatory, prebiotic, and anticancer properties. However, toxicity analyses still need to be carried out. Different doses of these *Gl*-1 or *Gl*-2 extracts were administered to Wistar rats for 14 days in a repeated dose oral toxicity study. We assessed the external clinical signs, biochemical parameters, liver and kidney tissues, injury and inflammation biomarkers, gene expression, inflammatory responses, proinflammatory mediators, and gut microbiota. *Gl* extracts had no significant adverse, toxic or harmful effects on male and female rats compared to the control groups. No injury or dysfunction were recorded in the kidney or liver, as there were no significant abnormal variations in organ weight, tissue histopathology, serum biochemical parameters (C-reactive protein, creatinine, urea, glucose, ALT and AST transaminases, TC, LDL-c, TG, HDL-c), urinary parameters (creatinine, urea nitrogen, albumin, the albumin-to-creatinine ratio, glucose), injury and inflammatory biomarkers (KIM-1/TIM-1, TLR4, and NF-кB protein expression; IL-1β, TNF-α and IL-6 gene expression), or the expression of genes linked to cholesterol metabolism (HMG-CoA, Srebp2, Ldlr). *Gl*-1 and *Gl*-2 extracts showed prebiotic effects on the gut microbiota of male and female Wistar rats. Bacterial diversity and relative bacterial abundance (BRA) increased, positively modulating the Firmicutes/Bacteroidetes ratio. The ASA (10 mM) added to the substrate used for mushroom cultivation changed properties and effects of the *Gl*-2 extract on Wistar rats. The no-observed-adverse-effect-level (NOAEL) was 1000 mg/kg body weight/day of *Gl*-1 or *Gl*-2 extracts. Clinical trials are recommended for further exploring the potential therapeutic applications of studied extracts.

## Introduction

Edible, functional, and medicinal mushrooms are becoming increasingly important in the global food system and for human health worldwide [1, 2]. The economic value of the genus *Ganoderma* has been estimated in excess of USD 5 billion annually [3], an industry increasing yearly. The type species, *Ganoderma lucidum* (Curtis) P. Karst. (Fungi, Polyporales, Ganodermataceae) *sensu lato* is a cosmopolitan medicinal mushroom cultivated on a large or small scale for producing a variety of commercial preparations, dietary supplements, and natural health products in many regions, particularly in Asia. This species, along with other closely related taxa of the complex [4], is gaining attention from the scientific community, as it contains diverse and potent bioactive compounds conferring specific functional and medicinal properties. Potent and pharmacologically active compounds have been found in *G*. *lucidum*, mainly polysaccharides (α-glucans, β-glucans), triterpenes, and polyphenols [5]. Specific pharmacological activities of α-glucans and β-glucans include antitumor and immunomodulatory effects, triterpenes induce apoptosis and anti-inflammatory responses, and polyphenols show antioxidant, free radical scavenging, and antiviral activity. Furthermore, positive effects and therapeutic applications of *G*. *lucidum* have been demonstrated, involving antitumor, immunomodulatory, antimicrobial, antioxidant, hypocholesterolemic, antihypertensive, anti-inflammatory, antithrombotic, antidiabetic, and hepatoprotective activities [6–8]. Most research works have been carried out using strains from East and Southeast Asia (China, Korea, Japan), where *G*. *lucidum* has been part of the traditional medicine, and to a lesser extent from Europe and the USA [5, 9–14].

The traditional use of *G*. *lucidum* for promoting health and longevity is well documented in Asian countries, involving better vital energy, strengthened cardiac function, increased memory, and antiaging effects [10]. *G*. *lucidum* has had a long history of use as safe traditional medicine for about 2000 years. This use has now been validated through the development of diverse bioactive compounds and products tested in various *in vivo* models (mice, rats, rabbits, dogs). Effective doses and dosage forms have been determined and recommended for human consumption, although not thoroughly subjected to postmarketing surveillance studies. Acute, subchronic, and genetic toxicity studies have shown no toxicity or adverse effects [15–23], insignificant side effects, or low toxicity at extremely high experimental doses, *e*.*g*., LD_50_ 38.3 g/kg i.g. [10, 24, 25]. However, thorough elucidation of molecular mechanisms of action of *G*. *lucidum*, through gene expression, genomic, and biochemical analyses, is needed to promote further clinical research in phases II-IV [26–31]. This is difficult, as the structure and composition of *G*. *lucidum* compounds and products vary according to the geographical origin of the strain, cultivation methods, the stage of development (basidiocarp, spores, mycelium), extraction procedures, standardization techniques, product development, and commercial batch. Complete safety analyses of therapeutic dosages from bioactive compounds of *G*. *lucidum* would allow determining their long-term effects, bioavailability, pharmacokinetics, pharmacodynamics, efficacy, and effectiveness after administration, as well as their interactions with other drugs.

Our studies on Mexican genetic resources of *G*. *lucidum*, known as “repisas” (brackets) by indigenous and peasant communities, have shown that they represent a new promising source of bioactive compounds exerting antioxidant, antibacterial, hypocholesterolemic, anti-inflammatory, prebiotic, and anticancer properties [2, 32–34]. Standardized extracts, characterized in terms of their complex nutrient composition, are effective at low safe concentrations using *in vitro* and *in vivo* models, and exploratory clinical trials. We have developed a novel strategy to modulate the functional and medicinal properties of *G*. *lucidum* extracts through acetylsalicylic acid (ASA) in the cultivation substrate [2, 32, 35]. Products derived from these studies are in progress for therapeutic use treating hypercholesterolemia, metabolic syndrome, cellular inflammation, and modulation of the gut microbiota. In this study, we assessed the safety of these standardized extracts of *G*. *lucidum* performing, for the first time in the Mexican genotype, a repeated dose oral toxicity test using an *in vivo* model, based on standard protocols [36, 37], FDA guidelines [38], and principles of good laboratory practices. We administered different doses of *G*. *lucidum* extracts to male and female Wistar rats, according to a dietary approach. Their effects on external clinical variables, biochemical parameters, biomarkers, liver, kidney, and the gut microbiota were analyzed.

## Materials and methods

### Standardized extracts and chemical characterization

#### Cultivation and harvesting of *Ganoderma lucidum* (*Gl*)

The CP-145 strain of *G*. *lucidum* (Curtis) P. Karst. was isolated by tissue culture from a wild basidioma growing on a dead tree in the State of Morelos, Mexico (2,300 m altitude). This strain is deposited at the Centre for Biotechnology of Medicinal, Functional, and Edible Mushrooms (CB-HCFM), CP, Mexico. The gene sequence of the ITS1-5.8S-ITS2 rDNA from the CP-145 strain is deposited at the GenBank (www.ncbi.nlm.nih.gov/genbank/), with the accession number LN998989. Wheat grain spawn was prepared using this strain according to standard methods. Oak (*Quercus acutifolia* Née) sawdust was introduced into polypropylene plastic bags (51 x 21 cm) with a microfilter of 0.5 microns (3 x 8 cm) for allowing gas exchange (CB-HCFM, Mexico). Two groups of bags were prepared for mushroom cultivation, the control and treatments, according to the methodology described previously [1]. Distilled water (1300 mL) was added to each control bag containing 1000 g of dry oak sawdust, and mixed homogeneously. A solution of acetylsalicylic acid (ASA, 10 mM; Sigma-Aldrich, USA) in distilled water (1300 mL) was added to every treated bag containing 1000 g of dry oak sawdust, and mixed homogeneously. All bags (2 kg wet substrate per bag) were sterilized at 121°C for 90 min. After cooling, under aseptic conditions, sterilized substrates were inoculated by uniform mixing at a rate of *ca*. 50 g of CP-145 spawn per kg of fresh substrate weight (5%, w/w). Inoculated plastic bags were sealed, and placed on shelves for incubation in the dark (24°–25°C). Bags were moved to the fruiting room when fully colonized by the mushroom mycelium, maintaining constant conditions of temperature (20°–25°C), relative humidity (60–70%), and natural ventilation. The roof had two rows of transparent plastic sheets, which allowed indirect daylight (about 12 h) during the fruiting cycle. Average mycelial colonization of the substrates and differentiation of first primordia took 75 days in control bags and 66 days in bags treated with ASA, whereas average complete development of mature basidiomata lasted for 169 and 158 days, respectively. Mature basidiomata from each bag were harvested, cut into slices (*ca*. 1–2 cm), dried at 40°C in a forced air drying oven (SMO28-2, Shel Lab, USA) for five days, and stored at –80°C inside plastic bags until use [1].

#### Standardized mushroom extracts

Dried mushroom slices were chopped in a blender, and 10 g of the product was placed in a filter paper (8 μm) bag for maceration (24 h). Hydroalcoholic extracts, 32% v/v (alcohol:water), were obtained according to a previous patent (MX322035-B) [35]. Mushroom extracts were concentrated to 10 mL in a rotary evaporator (HS-2000NS, Hahn Shin Scientific, South Korea) at 19°C. They were then filter-sterilized (0.45 μm, Merck Millipore, Mexico), and stored at 4°C until use. In this study, standardized extracts of *G*. *lucidum* cultivated on the control substrate were labeled as *Gl*-1, whereas those cultivated on the treated substrate (ASA, 10 mM) were labeled as *Gl*-2. These extracts are compared in a repeated dose oral toxicity study, as *Gl*-1 and *Gl*-2 have been shown to exert different functional and medicinal properties [2, 32, 34].

#### Nutrient composition of *Gl* extracts

Standardized extracts of *G*. *lucidum* (*Gl*-1 and *Gl*-2) were previously analyzed according to standard protocols [32]. *Gl*-1 and *Gl*-2 contain carbohydrates (0.58%), glucans (15.96–17.01%, w/w), total protein (0.315–0.365%), total dietary fiber (0.10–0.15%), fat (0.01%), vitamins (B_1_, B_2_, B_3_, B_6_, B_12_, and D), minerals (calcium, copper, ion, magnesium, manganese, phosphorus, potassium, selenium, sodium, and zinc), several organic acids, and an energy content of 4 kcal/100 g extract. There are differences between nutrient profiles of *Gl*-1 and *Gl*-2 (S1 Table), including total protein, total dietary fiber, vitamins (B_1_, B_2_, B_3_, and B_12_), and minerals (copper, iron, magnesium, manganese, phosphorus, potassium, sodium, and zinc). These differences are due to the addition of ASA (10 mM) to the substrate, as all other variables remained constant during mushroom cultivation (*i*.*e*., species, strain, substrate, and environmental conditions).

### Animals and repeated dose toxicity test

The non-clinical safety testing of *Gl* extracts was performed according to a repeated dose oral toxicity test, adapted from standard protocols [36, 37], and FDA guidelines [38], considering national regulatory policy, research advances on *Ganoderma* and studies to be performed at the institution (CP). Thirty male (M) and thirty female (F) Wistar rats (*Rattus norvegicus*), 8–9 weeks old and weighing 150–200 g, were obtained from the animal facility at the INCMNSZ, Mexico City. Rats were housed at 23±2°C, with a relative humidity of 45–55%, on a 12-hour light/12-hour dark cycle. Male and female rats were randomly and independently assigned to ten experimental groups of six animals each (three males, three females), which were treated with one type of mushroom extract (five groups for *Gl*-1, five groups for *Gl*-2). The basic diet for all experimental groups was the AIN-93 [39], whose ingredients per 1000 g of diet are as follows: L-cysteine (3.0 g), choline (2.5 g), vitamins (10 g), cellulose (50 g), minerals (35 g), soybean oil (70 g), starch (397.5 g), dextrin (132 g), saccharose (100 g), and casein (200 g). Extracts were administered orally, homogeneously mixed with AIN-93 after 8 hours of fasting state. We compared the toxicity of *Gl*-1 or *Gl*-2 extracts, considering four different doses. For oral administration of *Gl*-1 and *Gl*-2 extracts in male (M) and female (F) rats, we established the following independent groups as described in the S2 Table: Ctrl: Control diet (AIN-93); 300 mg/kg *Gl* extract dose; 1000 mg/kg *Gl* extract dose; 2000 mg/kg *Gl* extract dose; and 5000 mg/kg *Gl* extract dose. Each group, one rat per cage, was fed *ad libitum* for 14 days, ensuring water, making sure that the full dose of *Gl*-1 or *Gl*-2 extracts had been eaten per day according to experimental diets. Food consumption was recorded daily, whereas body weight was registered twice weekly. Animals were observed closely every day for signs of toxicity. During the last week, feces were collected daily for gut microbiota analysis. At the end of the study, all sixty rats were deprived of food and water for 12 h before blood sampling, anesthetized with 3% sevoflurane, and then sacrificed on day 15. Blood was collected via the portal vein. The serum was obtained by centrifugation at 1000*g* for 10 min and stored at –70°C until analysis. Liver and kidney were rapidly excised, weighed, frozen in liquid N_2_, and stored at –70°C. Approval for this experiment was issued by the Animal Research Regulation Committee of the INCMNSZ, Mexico City (Permit number: FNU-1856-16/17-1). All researchers involved had advanced training in animal care and good laboratory practices. Humane endpoints considered in this study were body weight loss greater than 20%, severely compromised posture, ruffled fur, and/or indicators of pain.

### Serum biochemical parameters

Serum concentrations of total cholesterol (TC), glucose, low-density lipoprotein cholesterol (LDL-c), total triglycerides (TG), high-density lipoprotein cholesterol (HDL-c), C-reactive protein (CRP), creatinine, urea, alanine transaminase (ALT), and aspartate transaminase (AST) were measured using a COBAS C111 analyzer (Roche Diagnostics Ltd., Switzerland) [40].

### Urinary biochemical parameters

Urinary concentrations of glucose, urea nitrogen, creatinine, and albumin were assessed using a Beckman Coulter AU 5800 (Beckman Coulter Inc., Brea, CA, USA). The albumin-to-creatinine ratio (ACR) was calculated by dividing albumin concentration in milligrams by the creatinine concentration in grams.

### Histopathological analysis

Liver and kidney samples from male and female Wistar rats were dissected and immediately fixed with ice-cold paraformaldehyde (4% w/v), dissolved in phosphate buffer, and subsequently dehydrated and embedded in paraffin. Two sections (4 μm) per block were then stained with hematoxylin and eosin [41], and analyzed under a microscope (Leica DM750, Germany).

### KIM-1/TIM-1 protein in kidney tissue

This protein was determined in kidney tissue by KIM-1/TIM-1 Rat ELISA Kit (no. ab119597), according to the manufacturer’s instructions (Abcam, USA).

### RNA extraction

Total RNA was isolated from frozen rat liver using the TRIzol reagent (Invitrogen, USA), according to the manufacturer’s instructions. RNA was quantified using the Nanodrop 2000 spectrophotometer (Thermo Scientific, USA).

### Quantification of gene expression by reverse transcription polymerase chain reaction (RT-PCR)

RNA was reverse-transcribed to cDNA using Moloney murine leukemia virus (MMLV) reverse transcriptase (Invitrogen, USA). For real-time PCR analyses of cDNA from different samples, amplifications were performed using the SYBR Green System in a Light Cycler 4800 thermal cycler system (Roche Diagnostics Ltd., Switzerland). The primers used were targeted to HMG-CoA, Ldlr, Srebp2, IL-6, TNF-α, and IL-1β, which are described in S3 Table. The ribosomal protein S18 (RPS18) was used as a housekeeping gene. Expression values were obtained as the relative expression of the target gene minus the constitutively expressed RPS18 gene [relative expression = 2—(Ct, Target gene—Ct, Reference gene)]. Primers for PCR amplification were designed using the Primer3 program (Howard Hughes Medical Institute, USA). Assays for each gene were performed in triplicate.

### Western blot analysis

Proteins were extracted using RIPA (Radioimmunoprecipitation Assay) lysis buffer [1% IGEPAL CA-630, 0.5% sodium deoxycholate, 0.1% sodium dodecyl sulfate, 1 mM sodium fluoride, 2 mM sodium orthovanadate and complete protease inhibitor cocktail tablets (Roche Applied Science, Germany), all dissolved in phosphate-buffered saline]. The protein concentration was measured using the Lowry method with the DC Protein Assay (Bio-Rad, USA). Total protein (40 μg) was heated for 5 min in Laemmli sample buffer (Bio-Rad, USA) and loaded on 10–12% polyacrylamide gels, separated by sodium dodecyl sulfate-polyacrylamide gel electrophoresis and transferred to a polyvinylidene difluoride membrane. The protein loaded into each well of the gel was 40 μg, and the protein standard marker was the PageRuler Plus (no. 26620, ThermoFisher Scientific, USA). The gel was run 30 min at 75 V, and then 90 min at 100 V, before transfer to the polyvinylidene difluoride membrane. Blots were blocked for 1 h at room temperature with 5% blotting grade blocker nonfat dry milk (Bio-Rad, USA), and incubated overnight at 4°C with the following primary antibodies: GAPDH (G-9) (no. sc-365062, dilution 1:1500), NF-кB p65 (no. sc-372, dilution 1:2000), and TLR4 (no. sc-293072, dilution 1:10000), which were obtained from Santa Cruz Biotechnology (SCB, USA). Appropriate secondary antibodies were also obtained from SCB. The blots were then incubated with anti-rabbit or anti-goat immunoglobulin G antibody conjugated with horseradish peroxidase (SCB, USA), diluted 1:3500. Blots were developed by the enhanced chemiluminescence method with Immobilon Western Chemiluminescent HRP substrate (Millipore, USA), and the blot image was recorded through the ChemiDoc MP imaging system (Bio-Rad, USA). The samples were analyzed three times in independent blots. Semiquantification of bands was carried out by optical densitometry and analyzed using the ImageJ digital imaging processing software (ImageJ 1.48v, NIH, USA). The expression of each protein analyzed was normalized with GAPDH.

### Gut microbiota analysis

Fecal samples from male and female rats were immediately collected and frozen at –70°C. DNA extraction was performed using the QIAamp DNA Stool Mini Kit (Qiagen, USA), according to the manufacturer’s instructions. Variable regions 3–4 of the 16S rRNA gene were amplified using specific forward and reverse primers 5ˈ–3ˈ and 3ˈ–5ˈ, respectively, containing the Illumina adapter overhang nucleotide sequences. PCR was performed following the method described previously [32]. According to the manufacturer’s instructions, sequencing was performed on the Illumina MiSeq platform (MiSeq Reagent Kit V.3, 600 cycles) to generate paired-end reads 300 bases in length for each direction. Overlapping paired-end reads were merged using fastq-join and were processed with QIIME V.1.9 [42]. Only Illumina reads with an average score above 20 were retained for further analysis. Low-quality and chimeric sequences were removed. Reads were assigned to operational taxonomic units (OTUs) using Usearch V5.2.236 [43] at a 97% similarity threshold. OTUs were checked in the RDP database. In this way, 99.9%, 99.7%, 99.5%, 90.4% and 89.9% of the reads were assigned to phylum, class, order, family and genus levels, respectively, based on a taxonomic reference database. The alpha diversity was calculated using the Shannon index, within-sample diversity was estimated at a rarefaction. Weighted and unweighted UniFrac distances were used to perform principal coordinates analysis (PCoA). Microbial sequence data were pooled for OTU comparison and taxonomic abundance analysis, but they were batch separated in PCoA to have clear PCoA figures. PCoA were created using Emperor, community diversity was determined by number of OTUs and beta diversity as measured by unweighted and weighted UniFrac distance. ANOSIM was used to determine statistically significant clustering of groups as a function of microbiota structure distances.

### Statistical analysis

Experiments were carried out in triplicate. Results are expressed as means ± SEM. Statistical significance was determined by one-way ANOVA (Tukeyˈs posthoc test), as appropriate, by using Prism statistical software (GraphPad Prism for Mac OSX v.7). Differences were considered significant at *p* < 0.05.

## Results

### Repeated dose oral toxicity test

We examined all male (M) and female (F) Wistar rats daily for external changes, patterns or symptoms during the study. Twenty different parameters were assessed, considering changes in unprovoked behavior and physical alterations. No deaths, visible side effects or allergic reactions were observed in any of the experimental groups during or at the end of the study after 14 days. There were no changes in all parameters studied. The only exception was that the rats that received the highest doses of *Gl*-1 or *Gl*-2 extracts (5000 mg/kg) were temporarily more active than the rest of the groups.

### Effects of *Gl*-1 and *Gl*-2 extracts on food intake, body weight gain, and organ weights of Wistar rats

#### *Gl*-1 extract

There was no significant difference in the food intake between groups of male or female Wistar rats that consumed the *Gl*-1 extract at different experimental doses, as compared to the controls (Table 1). There were no significant differences between treatments and control groups regarding the average weight gain and organ weights (liver, kidney) in male or female rats at the end of the study (Table 1).

**Table 1:** Average data from food intake, weight gain, liver and kidney weight, and serum biochemical parameters of male (M) and female (F) Wistar rats studied, after 14 experimental days. Rats were fed with the control diet and different doses of the standardized *Gl*-1 extract from *Ganoderma lucidum*.

**Parameter**	**Male rats (*Gl*-1 extract doses)**					
	**Ctrl *Gl*-1M**	**300 mg/kg *Gl*-1M**	**1000 mg/kg *Gl*-1M**	**2000 mg/kg *Gl*-1M**	**5000 mg/kg *Gl*-1M**	***p***
Food intake (g/day)	25.53±1.79	25.33±1.84	23.80±1.00	23.27±0.58	23.30±0.86	0.609
Weight gain (g)	91.25±1.29	84.67±2.60	83.00±2.30	84.00±1.73	79.67±4.84	0.139
Initial weight (g)	229.67±5.78	232.00±14.11	228.67±14.31	234.67±6.43	227.33±18.17	0.994
Final weight (g)	320.92±1.15	316.67±20.12	311.67±23.31	318.67±19.15	307.00±17.16	0.980
Liver weight (%)	3.95±0.12	4.14±0.12	4.20±0.18	4.29±0.10	4.46±0.34	0.494
Kidney weight (%)	0.89±0.03	0.89±0.04	0.95±0.01	0.94±0.01	0.93±0.06	0.706
Cholesterol (mg/dL)	50.96±1.46	51.05±2.99	52.27±1.54	57.25±0.48	52.48±1.14	0.140
Glucose (mg/dL)	158.40±2.03	168.40±1.40	157.10±5.22	171.40±3.21	157.00±2.20	0.018
LDL-c (mg/dL)	9.60±0.52^ab^	9.05±0.47^ab^	7.14±0.60^b^	10.63±0.55^a^	10.59±0.55^a^	0.006*
TG (mg/dL)	64.37±1.84	74.50±2.42	74.42±4.69	71.89±2.17	76.66±3.13	0.110
HDL-c (mg/dL)	36.98±3.93^b^	54.36±6.31^a^	50.60±2.02^ab^	57.93±1.23^a^	47.29±0.68^ab^	0.015*
CRP (mg/L)	0.12±0.01	0.11±0.01	0.11±0.01	0.12±0.01	0.10±0.01	0.675
Creatinine (mmol/L)	0.27±0.03^ab^	0.23±0.03^b^	0.10±0.00^c^	0.15±0.02^bc^	0.11±0.01^c^	0.002*
Urea (mmol/L)	5.87±0.44	6.48±0.46	5.14±0.20	5.81±0.48	6.22±0.46	0.293
ALT (U/L)	74.55±2.62^a^	63.00±3.13^ab^	76.02±0.76^a^	69.57±3.69^ab^	58.20±3.63^b^	0.007*
AST (U/L)	245.90±0.25^a^	220.2±8.89^abc^	203.80±15.31^b^	243.10±8.42^ab^	181.90±0.69^c^	0.002*
**Parameter**	**Female rats (*Gl*-1 extract doses)**					
	**Ctrl *Gl*-1F**	**300 mg/kg *Gl*-1F**	**1000 mg/kg *Gl*-1F**	**2000 mg/kg *Gl*-1F**	**5000 mg/kg *Gl*-1F**	***p***
Food intake (g/day)	16.80±0.50	18.00±0.11	17.20±1.10	15.83±0.68	16.23±1.19	0.425
Weight gain (g)	29.67±2.60	32.00±0.57	30.00±2.51	28.00±3.05	28.67±1.66	0.766
Initial weight (g)	182.00±9.16	180.67±3.28	185.00±5.13	165.33±3.48	184.00±3.21	0.135
Final weight (g)	211.67±11.86	212.67±2.66	215.00±7.63	193.33±4.41	212.67±2.72	0.227
Liver weight (%)	3.47±0.25	4.31±0.21	4.09±0.54	4.26±0.15	4.05±0.54	0.565
Kidney weight (%)	0.91±0.11	0.85±0.02	0.88±0.02	0.87±0.07	0.91±0.01	0.939
Cholesterol (mg/dL)	51.60±1.44	53.67±0.32	51.26±3.68	54.26±3.36	46.72±0.71	0.249
Glucose (mg/dL)	163.60±6.85	151.60±0.91	165.70±7.54	160.80±4.54	160.40±3.63	0.429
LDL-c (mg/dL)	10.20±0.42^a^	5.95±0.31^b^	10.05±0.55^a^	8.11±0.81^ab^	8.04±0.21^ab^	<0.0001*
TG (mg/dL)	74.86±2.43^ab^	80.21±2.55^ab^	70.96±0.66^b^	81.42±0.30^a^	80.83±2.20^a^	0.010*
HDL-c (mg/dL)	48.10±1.57^ab^	50.33± 0.43^ab^	60.36±0.80^a^	52.79±1.17^ab^	45.60±6.13^b^	0.040*
CRP (mg/L)	0.09±0.01	0.10±0.01	0.09±0.01	0.10±0.01	0.10±0.01	0.609
Creatinine (mmol/L)	0.27±0.03^a^	0.25±0.02^a^	0.20±0.00^ab^	0.23±0.03^a^	0.11±0.01^b^	0.007*
Urea (mmol/L)	6.49±0.19	6.16±0.51	6.42±0.51	6.71±1.08	6.76±0.14	0.950
ALT (U/L)	69.95±0.31^a^	72.47±1.40^a^	63.10±3.75^a^	44.75±0.60^b^	62.30±6.41^a^	0.001*
AST (U/L)	291.90±16.48^ab^	305.50±4.37^a^	251.40±10.68^b^	189.70±5.05^c^	260.30±4.53^b^	<0.0001*

Data are presented as mean ± SEM per experimental group, either male or female rats. Means in a row followed by different superscript letters indicate significant differences, *p* < 0.05. Values (*p*) correspond to one-way ANOVA from Tukey’s multiple comparison test. *Gl*-1: Extract of *G*. *lucidum* cultivated on the control substrate. The dose administered per experimental group is described in the materials and methods section. LDL-c: Low-density lipoprotein cholesterol. TG: Total triglycerides. HDL-c: High-density lipoprotein cholesterol. CRP: C-reactive protein. ALT: Alanine transaminase. AST: Aspartate transaminase.

* Significant difference.

#### *Gl*-2 extract

There were no significant differences in food intake and weight gain between treatments and control groups of male or female Wistar rats that consumed different doses of the *Gl*-2 extract (Table 2). Organ weights (liver, kidney) of male and female rats consuming the *Gl*-2 extract are shown in Table 2. There were no significant differences between treatments and control groups.

**Table 2:** Average data from food intake, weight gain, liver and kidney weight, and serum biochemical parameters of male (M) and female (F) Wistar rats studied, after 14 experimental days. Rats were fed with the control diet and different doses of the standardized *Gl*-2 extract from *Ganoderma lucidum*.

**Parameter**	**Male rats (*Gl*-2 extract doses)**					
	**Ctrl *Gl*-2M**	**300 mg/kg *Gl*-2M**	**1000 mg/kg *Gl*-2M**	**2000 mg/kg *Gl*-2M**	**5000 mg/kg *Gl*-2M**	***p***
Intake (g/day)	26.38±1.86	26.44±0.69	26.53±0.70	27.76±0.99	26.08±0.25	0.816
Weight gain (g)	62.33±1.20	67.67±3.52	70.33±4.05	71.33±3.75	68.67±1.85	0.339
Initial weight (g)	232.00±23.76	240.67±5.78	248.33±10.17	249.00±5.85	234.33±6.17	0.809
Final weight (g)	294.33±22.98	308.34±4.17	318.66±13.91	320.33±5.92	303±5.85	0.590
Liver weight (%)	3.47±0.24	3.80±0.12	3.81±0.06	4.01±0.08	3.83±0.18	0.264
Kidney weight (%)	0.85±0.03	0.84±0.01	0.83±0.01	0.84±0.01	0.90±0.01	0.157
Cholesterol (mg/dL)	62.89±2.32^ab^	51.00±3.09^c^	52.63±1.27^b^	68.20±1.60^a^	65.72±2.65^a^	<0.0001*
Glucose (mg/dL)	150.70±5.37	157.60±5.30	154.10±4.17	166.82±1.68	150.00±2.02	0.079
LDL-c (mg/dL)	8.34±0.47	9.32±0.07	9.84±0.89	8.41±0.67	10.08±0.53	0.207
TG (mg/dL)	86.80±2.26^a^	68.21±4.57^b^	95.52±3.69^a^	91.15±4.22^a^	98.59±1.85^a^	<0.0001*
HDL-c (mg/dL)	48.58±3.73^b^	43.76±0.93^b^	50.97±1.89^b^	65.14±3.02^a^	51.84±2.60^b^	0.002*
CRP (mg/L)	0.15±0.00	0.15±0.01	0.13±0.00	0.15±0.01	0.15±0.01	0.222
Creatinine (mmol/L)	0.20±0.00	0.23±0.03	0.27±0.03	0.20±0.00	0.23±0.03	0.769
Urea (mmol/L)	4.97±0.13	4.37±0.45	4.64±0.16	5.42±0.30	5.47±0.32	0.108
ALT (U/L)	42.67±3.05	40.20±3.46	39.80±3.98	41.20±1.64	40.45±1.53	0.958
AST (U/L)	180.40±4.15	186.00±11.00	139.30±18.88	163.00±1.65	179.30±11.98	0.084
	**Female rats (*Gl*2 extract doses)**					
	**Ctrl *Gl*-2F**	**300 mg/kg *Gl*-2F**	**1000 mg/kg *Gl*-2F**	**2000 mg/kg *Gl*-2F**	**5000 mg/kg *Gl*-2F**	***P***
Intake (g/day)	19.46±0.33	17.92±0.64	19.30±0.94	18.06±0.89	18.28±0.91	0.522
Weight gain (g)	30.00±1.15	31.33±1.20	30.33±1.85	29.67±2.33	27.33±1.85	0.595
Initial weight (g)	178.67±9.40	181.00±3.60	185.33±9.82	180.67±1.85	179.67±3.18	0.956
Final weight (g)	208.67±4.80	212.33±4.33	215.66±11.29	210.34±1.85	207.00±2.88	0.874
Liver weight (%)	3.36±0.06	3.43±0.08	3.65±0.02	3.13±0.21	3.30±0.23	0.256
Kidney weight (%)	0.84±0.01	0.85±0.01	0.89±0.01	0.88±0.01	0.84±0.03	0.332
Cholesterol (mg/dL)	67.62±3.36^ab^	69.38±1.56^a^	76.66±1.61^a^	59.07±2.29^b^	67.97±1.62^ab^	0.004*
Glucose (mg/dL)	155.70±3.50	154.00±2.46	155.00±6.52	149.40±2.41	150.00±5.45	0.777
LDL-c (mg/dL)	11.80±0.32	12.43±0.26	11.23±2.41	11.73±0.94	11.11±0.77	0.941
TG (mg/dL)	54.79±2.51	58.76±3.73	69.98±1.72	54.67±5.07	52.98±5.24	0.066
HDL-c (mg/dL)	57.23±4.09	60.24±1.90	61.80±0.23	59.23±3.68	54.67±1.00	0.410
CRP (mg/L)	0.12±0.01	0.12±0.01	0.12±0.01	0.11±0.01	0.13±0.01	0.849
Creatinine (mmol/L)	0.20±0.00	0.23±0.03	0.20±0.00	0.27±0.03	0.27±0.03	0.233
Urea (mmol/L)	3.59±0.02	3.29±0.14	3.71±0.33	4.44±0.34	4.20±0.28	0.059
ALT (U/L)	48.00±1.27^a^	40.30±3.27^ab^	35.70±0.92^b^	40.50±2.60^ab^	39.50±1.96^ab^	0.030*
AST (U/L)	182.10±12.87^ab^	153.10±9.00^b^	154.30±12.67^b^	184.30±1.24^ab^	215.00±3.37^a^	0.004*

Data are presented as mean ± SEM per experimental group, either male or female rats. Means in a row followed by different superscript letters indicate significant differences, *p* < 0.05. Values (*p*) correspond to one-way ANOVA from Tukey’s multiple comparison test. *Gl*-2: Extract of *G*. *lucidum* cultivated on the treated substrate (ASA, 10 mM). The dose administered per experimental group is described in the materials and methods section. LDL-c: Low-density lipoprotein cholesterol. TG: Total triglycerides. HDL-c: High-density lipoprotein cholesterol. CRP: C-reactive protein. ALT: Alanine transaminase. AST: Aspartate transaminase.

* Significant difference.

### Effects of *Gl*-1 and *Gl*-2 extracts on serum parameters of Wistar rats

#### *Gl*-1 extract

There were no significant differences in the concentrations of cholesterol (TC), glucose, CRP, and urea between groups of male or female Wistar rats fed with the control diet and different doses of the standardized *Gl*-1 extract (Table 1). Two parameters indicated positive trends in nearly all groups of male and female rats. Compared with the control group, HDL-c increased significantly in all groups of male and most groups of female rats. Conversely, creatinine decreased significantly in all groups of male and female rats. Total triglycerides (TG) showed no significant differences between groups studied of male rats, but groups of female rats showed up-and-down variability in comparison with the control group. A similar pattern was also recorded in groups of male rats for LDL-c. The concentration of ALT and AST transaminases showed decreasing trends in nearly all groups of male or female rats (Table 1).

#### *Gl*-2 extract

In parameters of glucose, LDL-c, CRP, creatinine, and urea, no significant differences were found between groups of male or female Wistar rats, fed with the control diet, and different doses of the standardized *Gl*-2 extract (Table 2). ALT and AST transaminases showed decreasing trends in nearly all groups of male or female rats (Table 2). Cholesterol (TC), total triglycerides (TG), and HDL-c parameters moved up and down showing significant differences, but not clear patterns (Table 2). However, these parameters were within normal reference values.

### Effects of *Gl*-1 and *Gl*-2 extracts on urinary parameters of Wistar rats

#### *Gl*-1 extract

Average data from urinary biochemical parameters of male and female Wistar rats, fed with the control diet and different doses of the standardized *Gl*-1 extract, are shown in Table 3. After 14 experimental days, glucose decreased significantly in most groups of male rats. A similar trend was recorded in the content of urea nitrogen and creatinine. In the case of albumin, there were no significant differences between the control (Ctrl *Gl*-1M: 3.69 mg/dL) and the other groups. The albumin-to-creatinine ratio (ACR) had a mild or moderate increase in male rats treated with the *Gl*-1 extract (127.4–279.0 mg/g), in comparison with the control group (123.8 mg/g).

**Table 3:** Average data from urinary biochemical parameters of male (M) and female (F) Wistar rats studied, after 14 experimental days. Rats were fed with the control diet and different doses of standardized *Gl*-1 or *Gl*-2 extracts from *Ganoderma lucidum*.

**Urinary parameter**	**Male rats (*Gl*-1 extract doses)**					
	**Ctrl *Gl*-1M**	**300 mg/kg *Gl*-1M**	**1000 mg/kg *Gl*-1M**	**2000 mg/kg *Gl*-1M**	**5000 mg/kg *Gl*-1M**	***p***
Glucose (mg/24 h)	6.67±0.33	3.93±0.06	5.33±0.66	7.33±0.88	6.00±1.15	0.062
Urea nitrogen (mg/24 h)	1175.00±171.80^a^	857.00±28.11^ab^	996.30±84.02^ab^	1315.00±144.30^a^	555.20±50.32^b^	0.005*
Creatinine (mg/dL)	29.81±4.86^ab^	23.43±0.73^ab^	28.49±3.11^ab^	31.55±2.62^a^	16.45±1.98^b^	0.030*
Albumin (mg/dL)	3.69±0.10	5.30±0.28	3.88±0.55	4.02±0.37	4.59±0.60	0.122
Albumin-to-creatinine ratio (ACR, mg/g)	123.8	226.2	136.2	127.4	279.0	
	**Female rats (*Gl*-1 extract doses)**					
	**Ctrl *Gl*-1F**	**300 mg/kg *Gl*-1F**	**1000 mg/kg *Gl*-1F**	**2000 mg/kg *Gl*-1F**	**5000 mg/kg *Gl*-1F**	***p***
Glucose (mg/24 h)	5.93±0.06^a^	7.50±0.28^a^	4.00±0.57^b^	6.50±0.86^a^	4.33±0.33^b^	0.003*
Urea nitrogen (mg/24 h)	1405.00±12.41^a^	1248.00±7.10^b^	448.50±1.44^d^	759.90±72.24^c^	469.40±4.96^d^	<0.0001*
Creatinine (mg/dL)	35.27±0.63^b^	47.43±1.48^a^	17.50±0.17^c^	28.90±3.11^b^	12.47±0.15^c^	<0.0001*
Albumin (mg/dL)	2.35±0.03^a^	0.38±0.02^c^	1.05±0.18^c^	2.73±0.31^a^	1.79±0.04^b^	<0.0001*
Albumin-to-creatinine ratio (ACR, mg/g)	66.6	8.0	140.0	94.5	143.5	
	**Male rats (*Gl*-2 extract doses)**					
	**Ctrl *Gl*-2M**	**300 mg/kg *Gl*-2M**	**1000 mg/kg *Gl*-2M**	**2000 mg/kg *Gl*-2M**	**5000 mg/kg *Gl*-2M**	***p***
Glucose (mg/24 h)	7.33±0.88^ab^	7.33±1.20^ab^	5.50±0.28^b^	8.00±1.00^ab^	10.00±0.57^a^	0.045*
Urea nitrogen (mg/24 h)	1472.00±184.00^a^	1282.00±126.00^ab^	907.60±108.50^b^	1483.00±13.17^a^	1372.00±86.78^ab^	0.034*
Creatinine (mg/dL)	47.20±3.11	47.38±3.98	37.77±2.42	45.91±2.39	51.38±3.81	0.118
Albumin (mg/dL)	0.46±0.03^b^	0.29±0.03^c^	0.46±0.05^b^	0.48±0.01^b^	0.64±0.02^a^	<0.001*
Albumin-to-creatinine ratio (ACR, mg/g)	9.7	6.1	12.2	10.5	12.5	
	**Female rats (*Gl*-2 extract doses)**					
	**Ctrl *Gl*-2F**	**300 mg/kg *Gl*-2F**	**1000 mg/kg *Gl*-2F**	**2000 mg/kg *Gl*-2F**	**5000 mg/kg *Gl*-2F**	***p***
Glucose (mg/24 h)	4.00±0.57	5.66±0.88	3.33±0.33	4.66±0.33	5.33±0.33	0.064
Urea nitrogen (mg/24 h)	559.10±30.48^b^	802.80±42.94^a^	705.00±58.89^ab^	755.20±14.32^a^	836.70±23.06^a^	0.003*
Creatinine (mg/dL)	35.58±3.16^abc^	43.89±1.97^a^	32.88±1.51^bc^	29.36±2.85^a^	41.73±1.41^b^	0.006*
Albumin (mg/dL)	0.24±0.01^b^	0.39±0.01^a^	0.16±0.01^b^	0.36±0.05^a^	0.37±0.03^a^	0.001*
Albumin-to-creatinine ratio (ACR, mg/g)	6.7	8.9	4.9	12.3	8.9	

Data are presented as mean ± SEM per experimental group, either male or female rats. Means in a row followed by different superscript letters indicate significant differences, *p* < 0.05. Values (*p*) correspond to one-way ANOVA from Tukey’s multiple comparison test. *Gl*-1: Extract of *G*. *lucidum* cultivated on the control substrate. *Gl*-2: Extract of *G*. *lucidum* cultivated on the treated substrate (ASA, 10 mM). The dose administered per experimental group is described in the materials and methods section.

In female Wistar rats (Table 3), there were two groups showing significantly lower urinary glucose concentrations (1000 mg/kg *Gl*-1F: 4.0 mg/24 h; 5000 mg/kg *Gl*-1F: 4.33 mg/24 h), whereas other two groups had slightly greater concentrations (300 mg/kg *Gl*-1F: 7.50 mg/24 h; 2000 mg/kg *Gl*-1F: 6.50 mg/24 h), in comparison with the control group (Ctrl *Gl*-1F: 5.93 mg/24 h). Urea nitrogen and creatinine decreased in comparisons with the control group in nearly all groups. Albumin concentration decreased in most groups compared to the control group. The ACR decreased or increased moderately in female rats treated with the *Gl*-1 extract, ranging from 8.0–143.5 mg/g, as compared to the control group (66.6 mg/g).

#### *Gl*-2 extract

Average data from urinary biochemical parameters of male and female Wistar rats, fed with the control diet and different doses of the standardized *Gl*-2 extract, are shown in Table 3. In most male rats, the glucose content was similar or significantly lower compared to the control group. Glucose increased significantly in the 5000 mg/kg *Gl*-2M group (10.0 mg/24 h). Urea nitrogen concentration was similar or decreased significantly in most male groups compared to the control group. There were no significant differences in creatinine concentration between the control and treatments. Albumin content was similar or significantly lower in most male groups compared to the control group. There was a mild decrease or increase of the ACR in male rats treated with the *Gl*-2 extract, varying from 6.1–12.5 mg/g, compared to the control group (9.7 mg/g).

Urinary glucose increased moderately in most groups of female Wistar rats (Table 3), in comparison with the control group. The exception was the group of 1000 mg/kg *Gl*-2F, which had a significantly lower urinary glucose level (3.33 mg/24 h). There was also a significant increase of urea nitrogen in all treated groups (705.0–836.70 mg/24 h), in comparison to the control group (559.10 mg/24 h). Creatinine increased in two groups (300 mg/kg *Gl*-2F: 43.89 mg/dL; 5000 mg/kg *Gl*-2F: 41.73 mg/dL), whereas it decreased in the rest of the groups (1000 mg/kg *Gl*-2F: 32.88 mg/dL; 2000 mg/kg *Gl*-2F: 29.36 mg/dL), in comparison with the control group (Ctrl *Gl*-2F: 35.58 mg/dL). Albumin increased significantly in most female groups, as compared to the control group. The exception was the group receiving 1000 mg/kg *Gl*-2F, in which albumin decreased significantly (0.16 mg/dL). The ACR decreased or increased mildly in female rats treated with the *Gl*-2 extract (4.9–12.3 mg/g), compared with the control group (6.7 mg/g).

### Effects of *Gl*-1 and *Gl*-2 extracts on hepatic and kidney tissues of Wistar rats

There were no significant differences between the control and treatments in the weight of liver or kidney from male or female Wistar rats, after 14 days of administering 300 mg/kg, 1000 mg/kg, 2000 mg/kg, or 5000 mg/kg of standardized *Gl*-1 and *Gl*-2 extracts (Tables 1 and 2).

**Fig 1:**
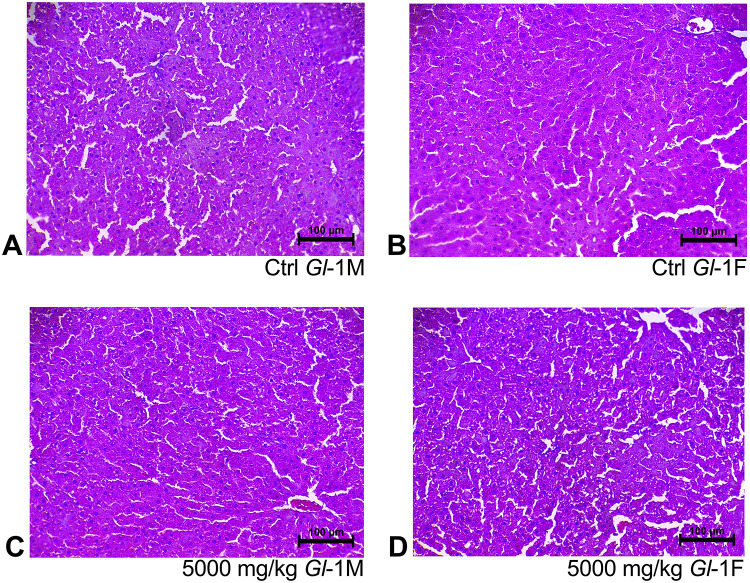
Hepatic tissue stained with hematoxylin and eosin, showed no liver damage in male (M) and female (F) Wistar rats after consuming the standardized *Gl*-1 extract of *Ganoderma lucidum* for 14 experimental days. A–B: Control (Ctrl) groups. C–D: Treated groups consuming the 5000 mg/kg bw/day dose.

**Fig 2:**
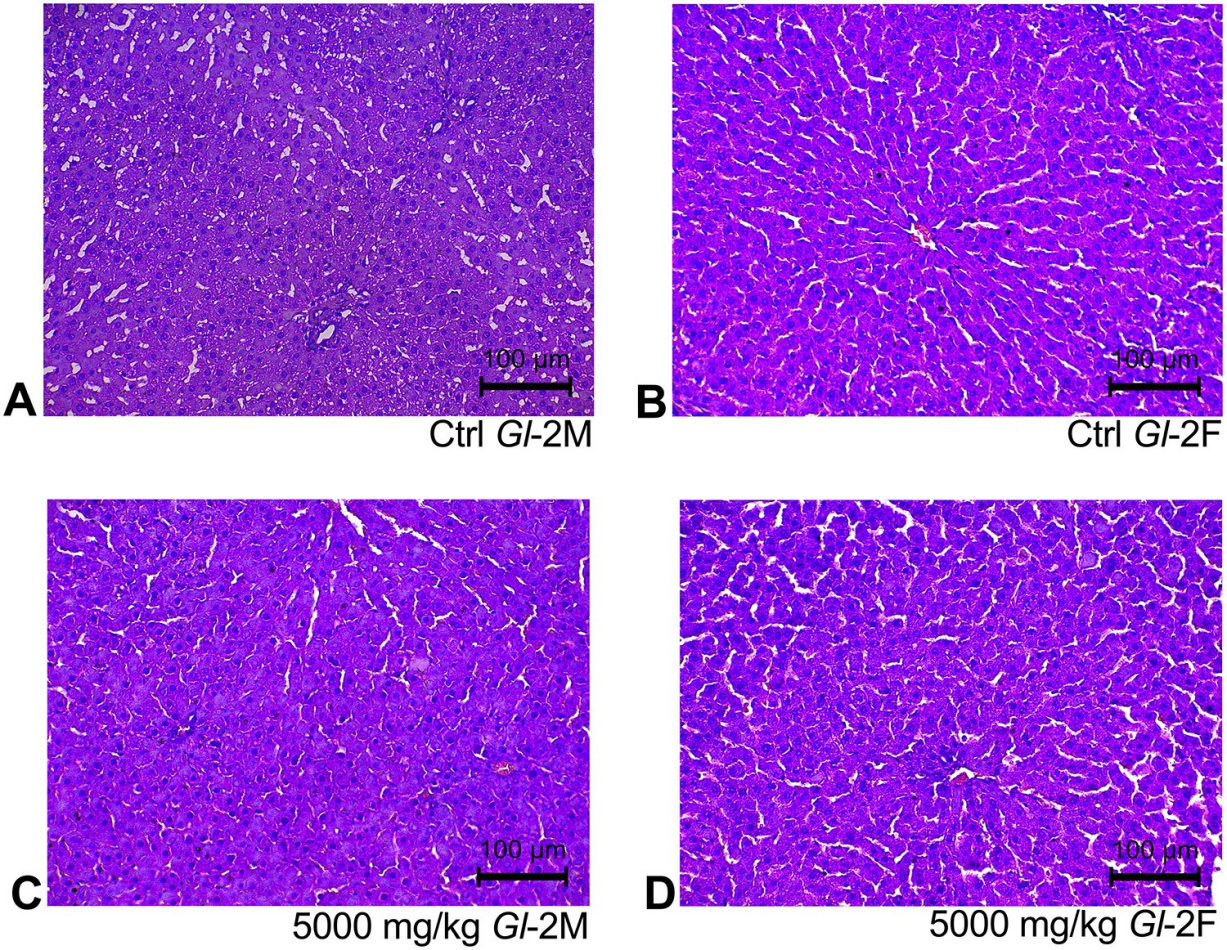
Hepatic tissue stained with hematoxylin and eosin, showed no liver damage in male (M) and female (F) Wistar rats after consuming the standardized *Gl*-2 extract of *Ganoderma lucidum* for 14 experimental days. A–B: Control (Ctrl) groups. C–D: Treated groups consuming the 5000 mg/kg bw/day dose.

**Fig 3:**
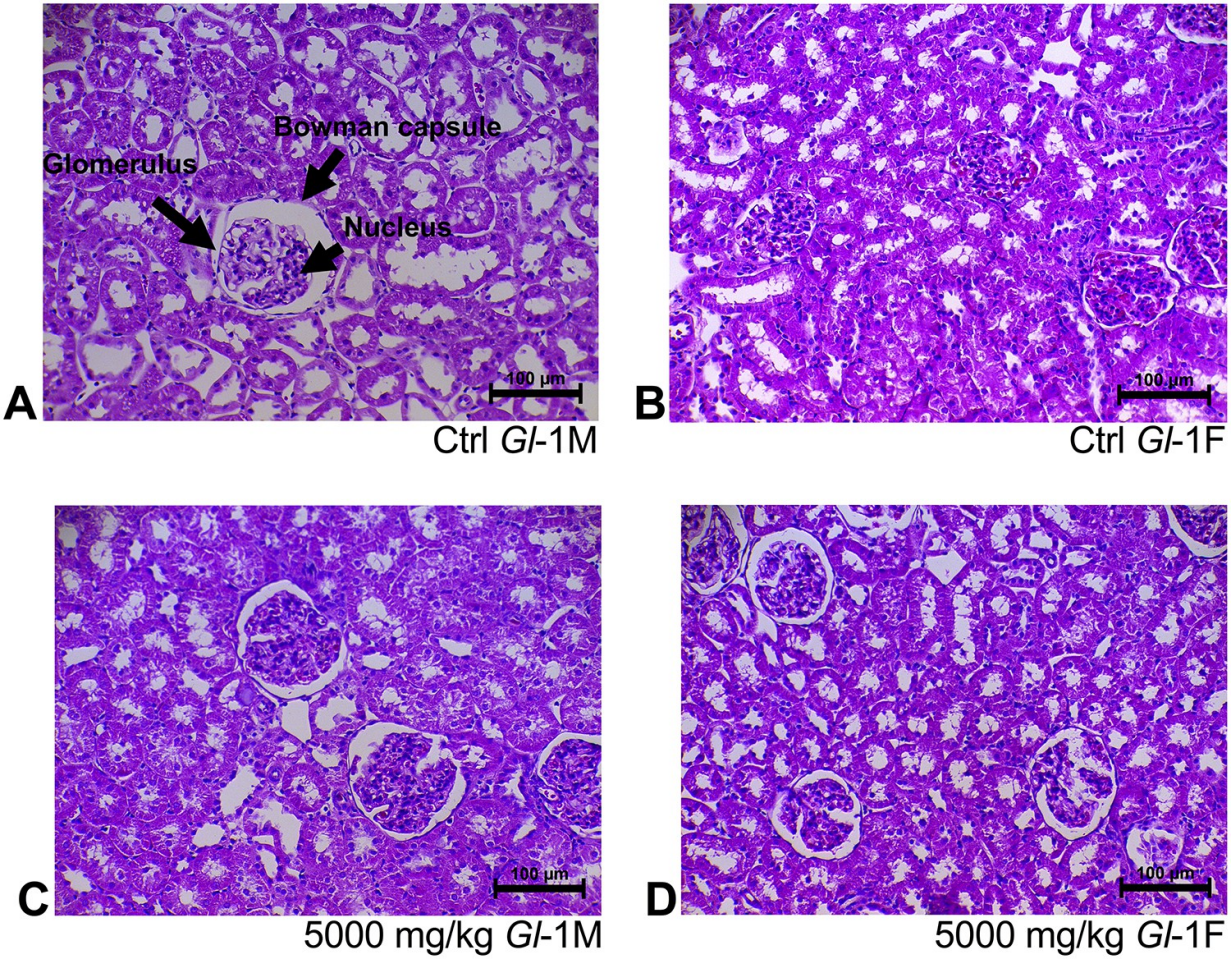
Kidney tissue stained with hematoxylin and eosin, showed no kidney damage in male (M) and female (F) Wistar rats after consuming the standardized *Gl*-1 extract of *Ganoderma lucidum* for 14 experimental days. A–B: Control (Ctrl) groups. C–D: Treated groups consuming the 5000 mg/kg bw/day dose.

**Fig 4:**
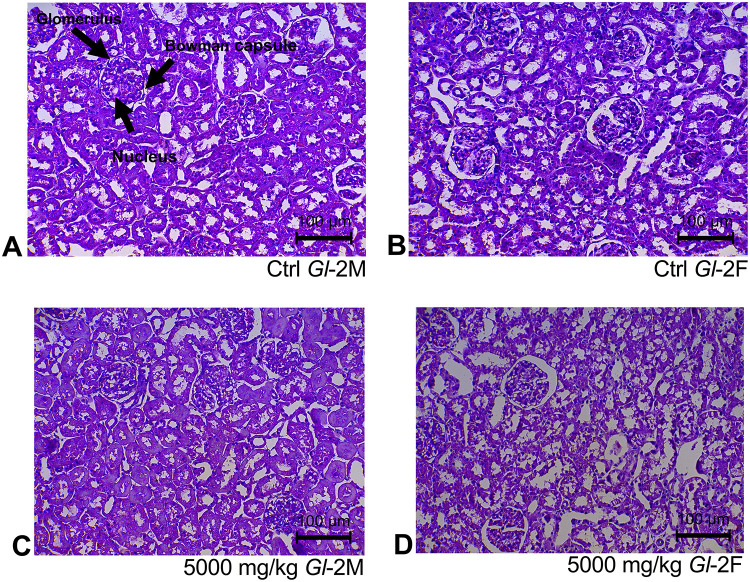
Kidney tissue stained with hematoxylin and eosin, showed no kidney damage in male (M) and female (F) Wistar rats after consuming the standardized *Gl*-2 extract of *Ganoderma lucidum* for 14 experimental days. A–B: Control (Ctrl) groups. C–D: Treated groups consuming the 5000 mg/kg bw/day dose.

Histological sections of liver tissue from male or female rats consuming different doses (300 mg/kg, 1000 mg/kg, 2000 mg/kg, 5000 mg/kg) of standardized *Gl*-1 and *Gl*-2 extracts for 14 days, showed normal cellular nuclei and uniform cytoplasm, as well as no damage or alterations of hepatocyte cytoarchitecture, as compared to the control group (Figs 1 and 2). This was also the case for renal tissue, as no damage was recorded in male or female rats fed with different doses (300 mg/kg, 1000 mg/kg, 2000 mg/kg, 5000 mg/kg) of standardized *Gl*-1 and *Gl*-2 extracts for 14 days. Normal renal tubules were observed (Figs 3 and 4), and no cellular damage (*e*.*g*., edema, necrotic proximal tubules, dead cells, glomerulosclerosis). There were no evident effects associated with extract doses consumed.

### Effects of *Gl*-1 and *Gl*-2 extracts on the KIM-1/TIM-1 protein of kidney tissue from Wistar rats

**Fig 5:**
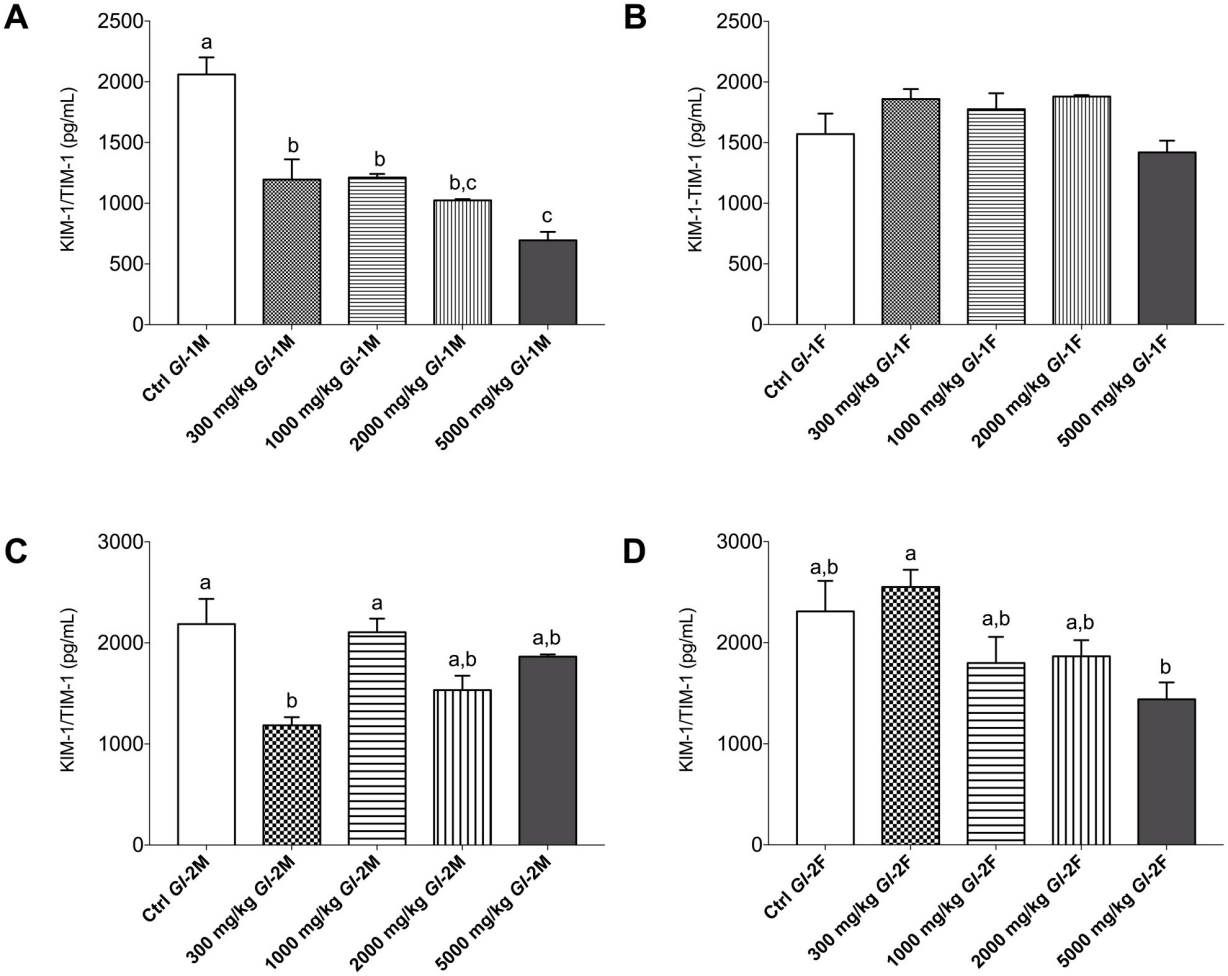
Renal KIM-1/TIM-1 protein (pg/mL) concentration in kidney tissue from the male (M) or female (F) Wistar rats fed with the control diet (Ctrl) and different doses of standardized *Gl*-1 or *Gl*-2 extracts of *Ganoderma lucidum* for 14 experimental days. Data are presented as mean ± SEM per experimental group. Different letters on a column indicate significant differences, *p* < 0.05. Values (*p*) correspond to one-way ANOVA from Tukey’s multiple comparison test. A–B: Administration of the *Gl*-1 extract. C–D: Administration of the *Gl*-2 extract. The dose was administered per experimental group as described in the materials and methods section.

KIM-1 is the most upregulated protein in the proximal tubule characterized by high sensitivity and specificity of kidney injury [44]. The consumption of the different doses of *Gl*-1 extract by male Wistar rats significantly decreased the amount of KIM-1/TIM-1 protein in renal tissue compared to the control group (Fig 5A). The greatest decreases of KIM-1/TIM-1 protein were recorded in the male rat group consuming 5000 mg/kg of *Gl*-1 extract, showing a decreasing pattern according to extract doses consumed. In female rats, however, there was no significant difference in the KIM-1/TIM-1 protein between experimental groups, although the greatest decrease was observed in the group consuming 5000 mg/kg of *Gl*-1 extract (Fig 5B). The KIM-1/TIM-1 protein in renal tissue also decreased significantly after male rats consumed the *Gl*-2 extract compared with the control group (Fig 5C). This was also the case in most female rats, except in the rat group consuming 300 mg/kg of *Gl*-2 extract, which showed a moderately increased amount of KIM-1/TIM-1 protein in renal tissue (Fig 5D).

### Effects of *Gl*-1 and *Gl*-2 extracts on the expression of other proteins associated to kidney injury of Wistar rats

**Fig 6:**
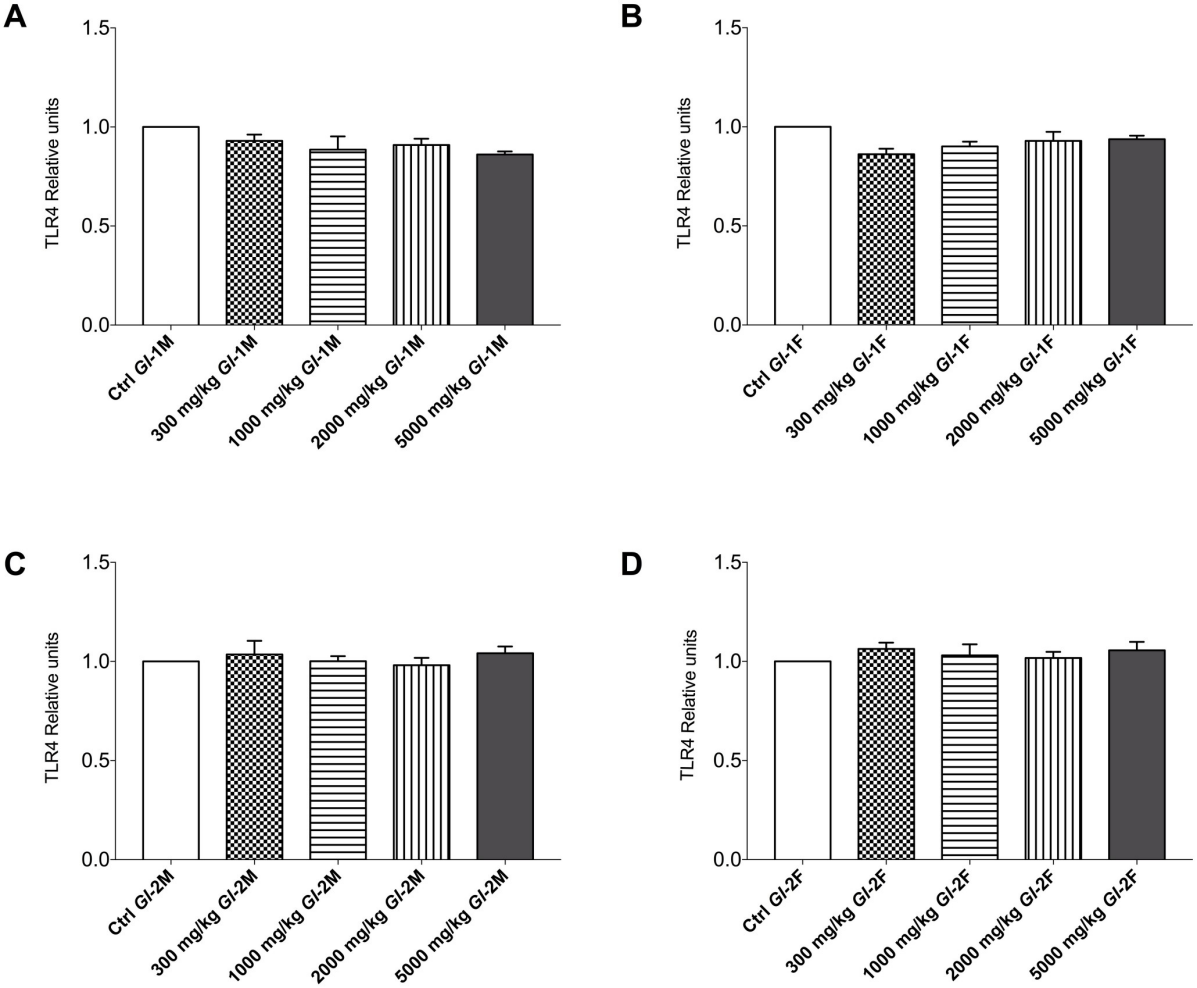
Renal TLR4 protein of kidney tissue from male (M) or female (F) Wistar rats fed with the control diet (Ctrl) and different doses of standardized *Gl*-1 or *Gl*-2 extracts of *Ganoderma lucidum* for 14 experimental days. Data are presented as mean ± SEM per experimental group. No significant differences were found (*p* < 0.05). Densitometry data were generated from Western blots shown in Fig 8. A–B: Administration of the *Gl*-1 extract. C–D: Administration of the *Gl*-2 extract. The dose administered per experimental group is described in the materials and methods section.

**Fig 7:**
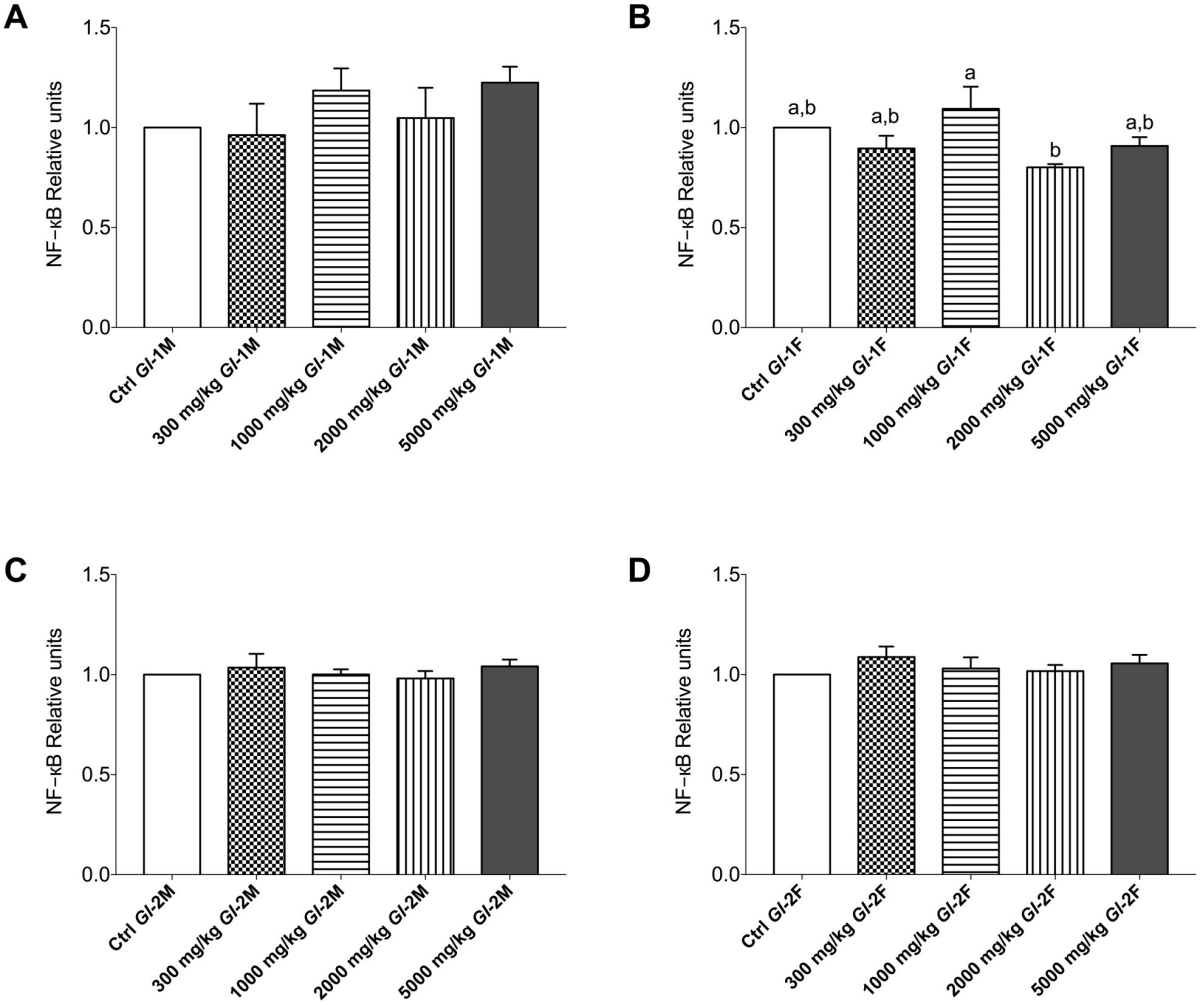
Renal NF-кB protein of kidney tissue from male (M) or female (F) Wistar rats fed with the control diet (Ctrl) and different doses of standardized *Gl*-1 or *Gl*-2 extracts of *Ganoderma lucidum* for 14 experimental days. Data are presented as mean ± SEM per experimental group. Different letters on a column indicate significant differences, *p* < 0.05. Values (*p*) correspond to one-way ANOVA from Tukey’s multiple comparison test. Densitometry data were generated from Western blots shown in Fig 8. A–B: Administration of the *Gl*-1 extract. C–D: Administration of the *Gl*-2 extract. The dose administered per experimental group is described in the materials and methods section.

**Fig 8:**
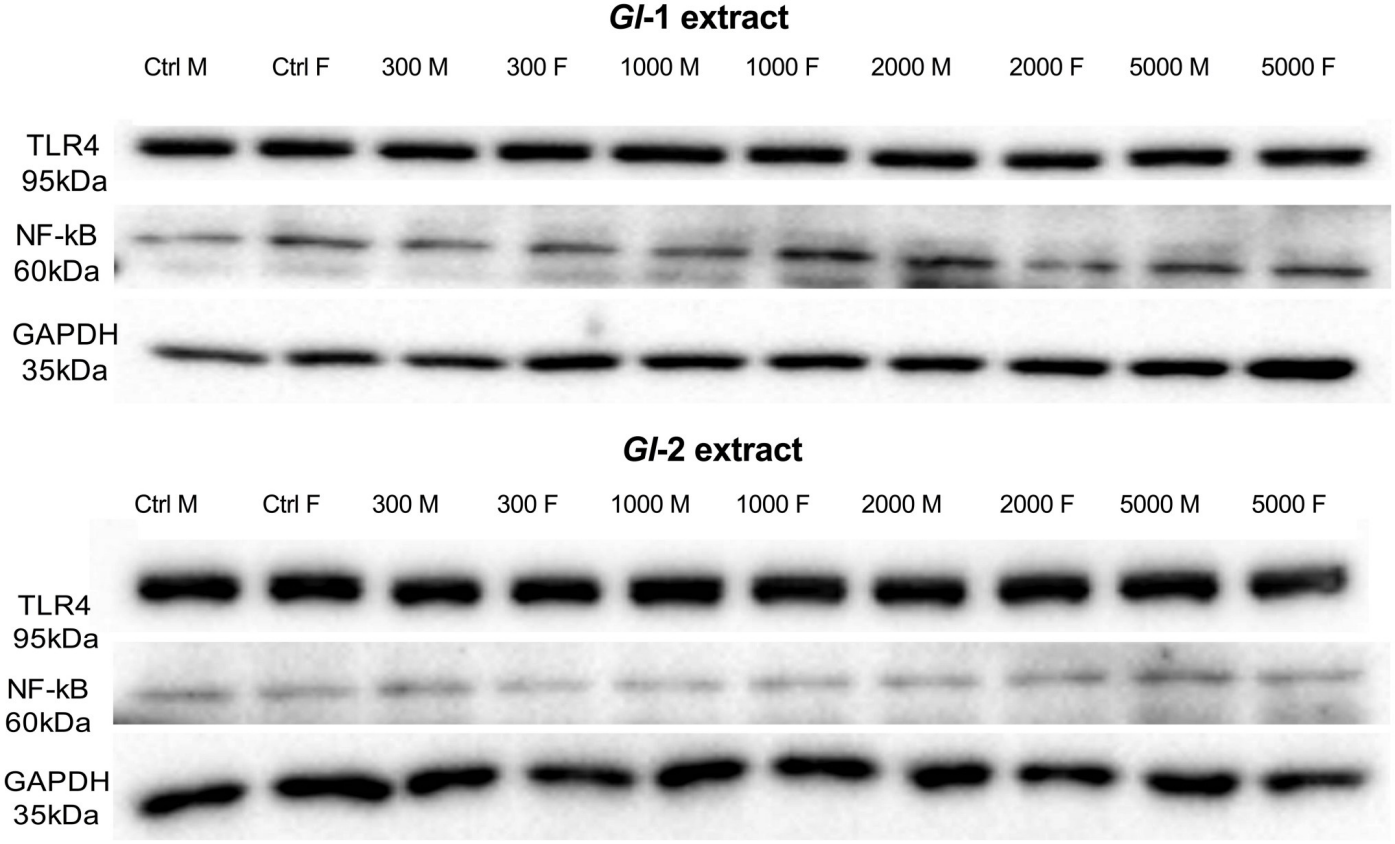
Western blot analyses of TLR4 and NF-кB expression in kidney tissue from male (M) or female (F) Wistar rats fed with the control diet (Ctrl) and different doses of standardized *Gl*-1 or *Gl*-2 extracts of *Ganoderma lucidum* for 14 experimental days. Corresponding densitometry data generated from Western blots are shown in Figs 6 and 7. The dose administered per experimental group is described in the materials and methods section.

The Toll-like receptor 4 (TLR4) and the transcription factor nuclear factor-kappa B (NF-кB) have a fundamental role in metabolic inflammation and kidney injury [45]. In this study, there were no significant variations in the protein abundance of TLR4 in kidney tissue from male and female Wistar rats consuming *Gl*-1 or *Gl*-2 extracts, compared with the control group. This was also the case for the NF-кB protein, whose abundance did not vary significantly in kidney tissue from male or female rats consuming the *Gl*-2 extract. However, slight variations in the NF-кB protein abundance, confirmed by Western blot, were recorded in kidney tissue from male and female rats consuming the *Gl*-1 extract. No specific variation pattern was associated with extract doses consumed (Figs 6–8).

### Effects of *Gl*-1 and *Gl*-2 extracts on the expression of genes related to cholesterol metabolism and inflammatory response in the liver of Wistar rats

#### Genes related to cholesterol metabolism

**Fig 9:**
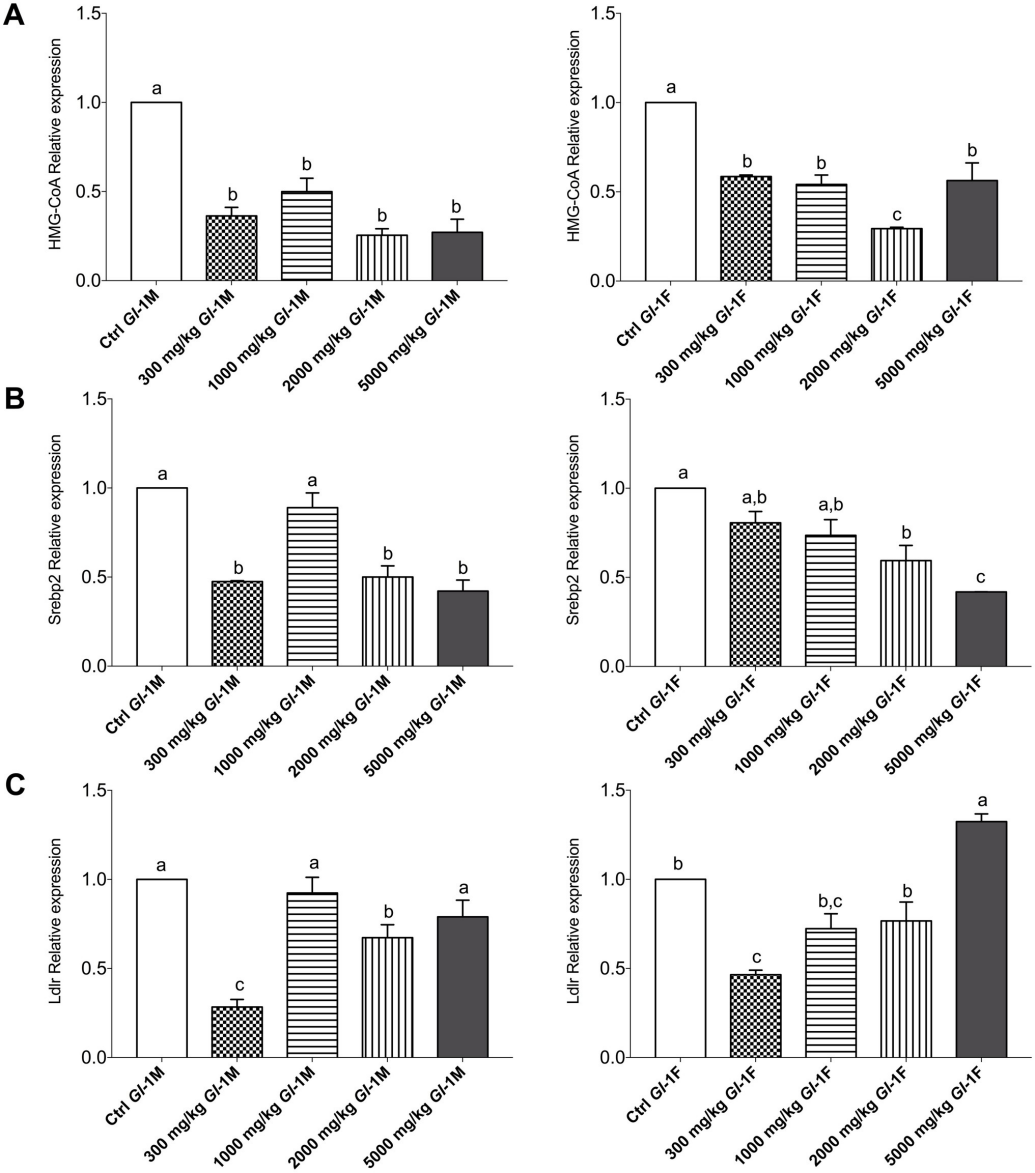
Expression of genes linked to cholesterol metabolism in liver tissue from male (M) or female (F) Wistar rats fed with the control diet (Ctrl) and different doses of the standardized *Gl*-1 extract of *Ganoderma lucidum* for 14 experimental days. A: HMG-CoA. B: Srebp2. C: Ldlr. Data are presented as mean ± SEM per experimental group. Different letters on a column indicate significant differences, *p* < 0.05. Values (*p*) correspond to one-way ANOVA from Tukey’s multiple comparison test. The dose administered per experimental group is described in the materials and methods section.

We analyzed the expression of these important genes, as *Gl* extracts have been shown to exert hypocholesterolemic effects [32]. Interestingly, although rats were not fed with a high-cholesterol diet, the *Gl*-1 extract significantly decreased gene expression of HMG-CoA in male and female Wistar rats, compared with the control group (Fig 9A). This was also the case for the expression of the Srebp2 gene, showing a decreasing dose-dependent pattern in female rats, after consumption of the *Gl*-1 extract (Fig 9B). The expression of the Ldlr gene also decreased in comparison with the control group, significantly in most cases, showing an increasing dose-dependent pattern associated to the *Gl*-1 extract dose, except for male rats consuming 1000 mg/kg. Only the female rats consuming 5000 mg/kg of the *Gl*-1 extract had a greater Ldlr gene expression than the control group (Fig 9C).

**Fig 10:**
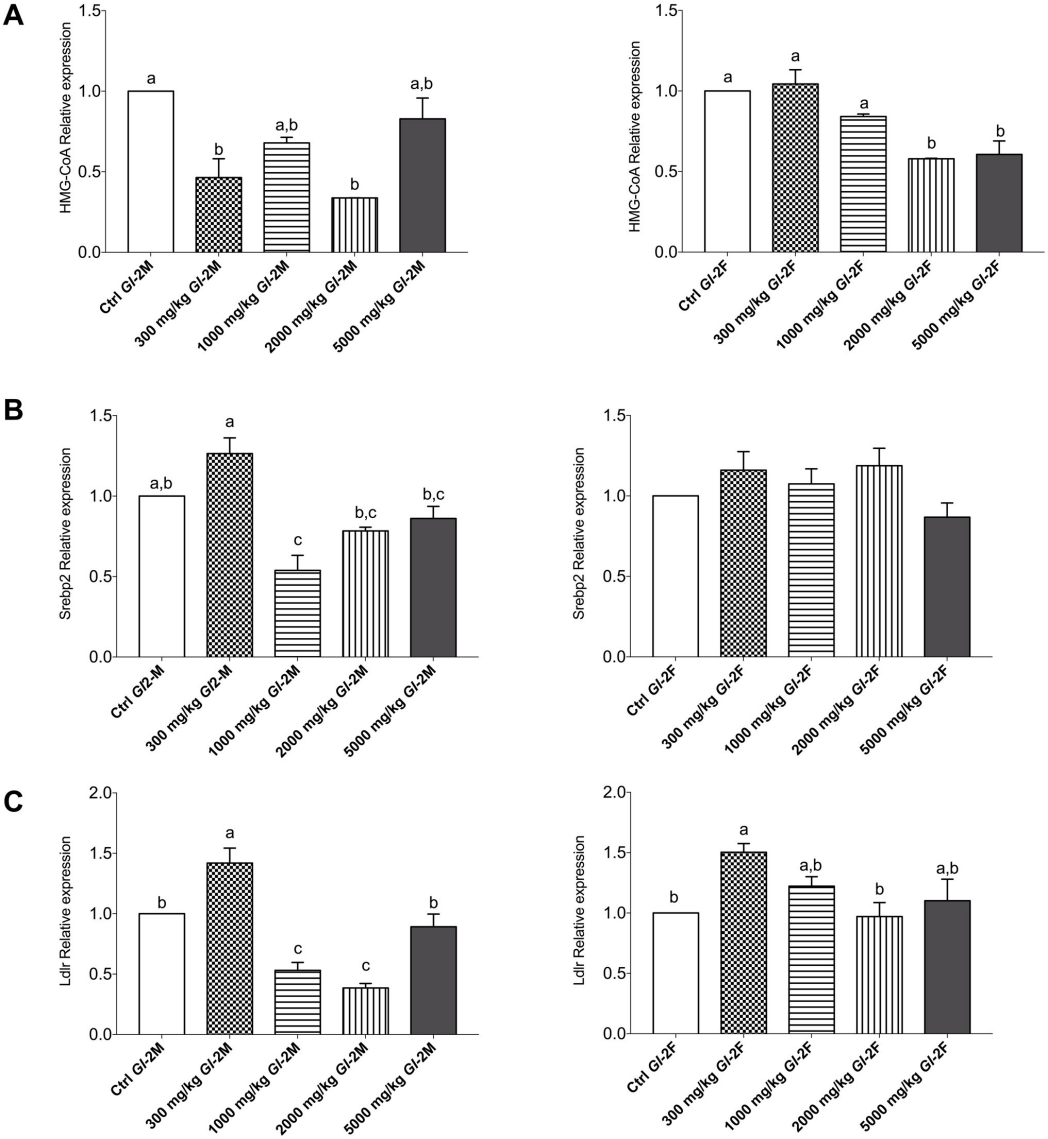
Expression of genes linked to cholesterol metabolism in liver tissue from male (M) or female (F) Wistar rats fed with the control diet (Ctrl) and different doses of the standardized *Gl*-2 extract of *Ganoderma lucidum* for 14 experimental days. A: HMG-CoA. B: Srebp2. C: Ldlr. Data are presented as mean ± SEM per experimental group. Different letters on a column indicate significant differences, *p* < 0.05. Values (*p*) correspond to one-way ANOVA from Tukey’s multiple comparison test. The dose administered per experimental group is described in the materials and methods section.

The consumption of the *Gl*-2 extract had similar effects on Wistar rats. The expression of the HMG-CoA gene was equal or decreased significantly in most groups of male or female rats compared with the control, showing no specific pattern of variation associated with the *Gl*-2 extract dose (Fig 10A). The expression of the Srebp2 gene was greater than the control in male rats consuming 300 mg/kg of the *Gl*-2 extract, while it decreased significantly in the other male rat groups. There were moderate up and down variations of Srebp2 gene expression in groups of female rats consuming the *Gl*-2 extract (Fig 10B). Similar was the Ldlr gene expression, as it was greater than the control in male rats consuming 300 mg/kg of the *Gl*-2 extract, while it decreased significantly in the other male rat groups. Up and down significant variations in the expression of the Ldlr gene were recorded in groups of female rats consuming the *Gl*-2 extract (Fig 10C).

#### Genes are involved in the inflammatory response

**Fig 11:**
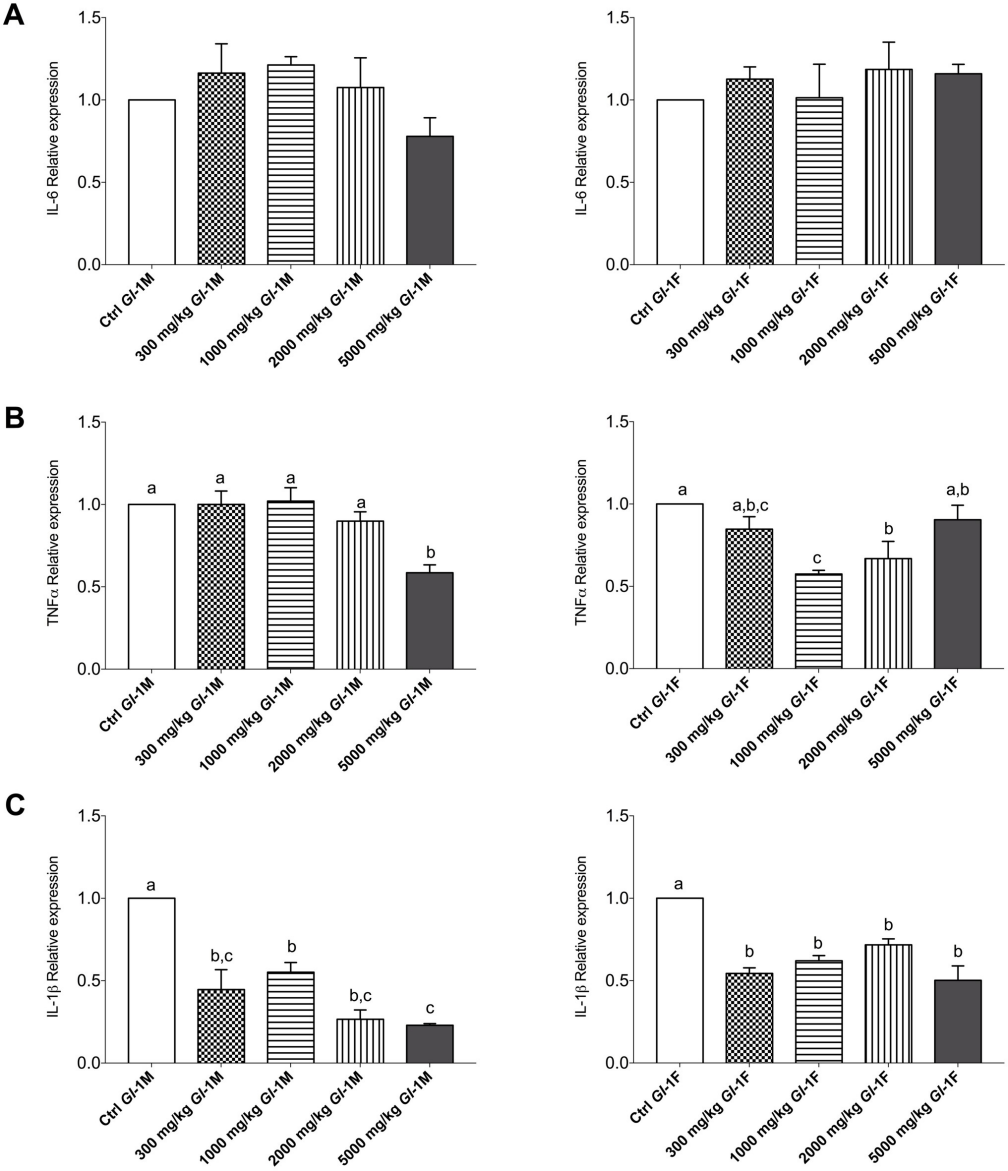
Expression of genes linked to inflammatory responses in liver tissue from male (M) or female (F) Wistar rats fed with the control diet (Ctrl) and different doses of the standardized *Gl*-1 extract of *Ganoderma lucidum* for 14 experimental days. A: IL-6. B: TNF-α. C: IL-1β. Data are presented as mean ± SEM per experimental group. Different letters on a column indicate significant differences, *p* < 0.05. Values (*p*) correspond to one-way ANOVA from Tukey’s multiple comparison test. The dose administered per experimental group is described in the materials and methods section.

Consumption of different doses of the *Gl*-1 extract by male and female Wistar rats had no significant effect on the expression of interleukin IL-6, compared to the control group (Fig 11A). In the case of TNF-α, compared to the control group, consuming the *Gl*-1 extract significantly decreased the expression of this gene in female rats (Fig 11B). However, in male rats this only happened in the group consuming the highest dose (5000 mg/kg). All groups of male or female rats consuming the *Gl*-1 extract showed significantly decreased IL-1β gene expression compared to the control group (Fig 11C).

**Fig 12:**
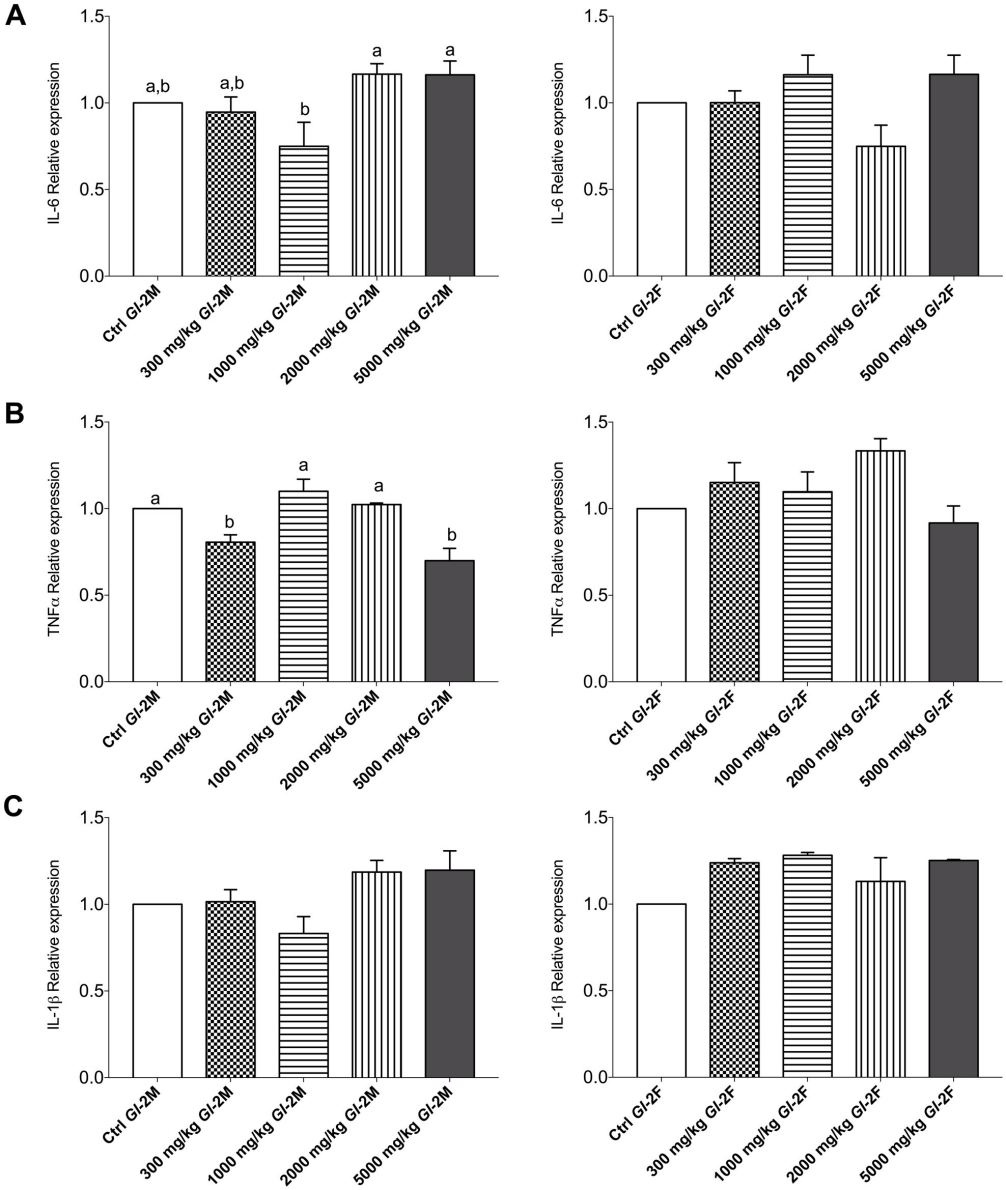
Expression of genes linked to inflammatory responses in liver tissue from the male (M) or female (F) Wistar rats fed with the control diet (Ctrl) and different doses of the standardized *Gl*-2 extract of *Ganoderma lucidum* for 14 experimental days. A: IL-6. B: TNF-α. C: IL-1β. Data are presented as mean ± SEM per experimental group. Different letters on a column indicate significant differences, *p* < 0.05. Values (*p*) correspond to one-way ANOVA from Tukey’s multiple comparison test. The dose administered per experimental group is described in the materials and methods section.

The consumption of the *Gl*-2 extract significantly decreased the expression of IL-6 gene in male Wistar rats consuming a dose of 1000 mg/kg, as compared to the control group. However, gene expression increased significantly in male rat groups consuming doses of 2000 mg/kg and 5000 mg/kg. By contrast, in groups of female rats, the consumption of *Gl*-2 extract did not significantly affect the expression of IL-6 gene (Fig 12A). The expression of TNF-α gene was equal or significantly decreased in male rat groups consuming 300 mg/kg and 5000 mg/kg doses, in comparison with the control group. The consumption of *Gl*-2 extract did not significantly affect the expression of TNF-α gene in groups of female rats (Fig 12B). The expression of the IL-1β gene was not affected significantly by the consumption of the *Gl*-2 extract in all male and female rat groups, compared with the control group (Fig 12C).

### Effects of different doses of *Gl*-1 and *Gl*-2 extracts on the gut microbiota of Wistar rats

**Fig 13:**
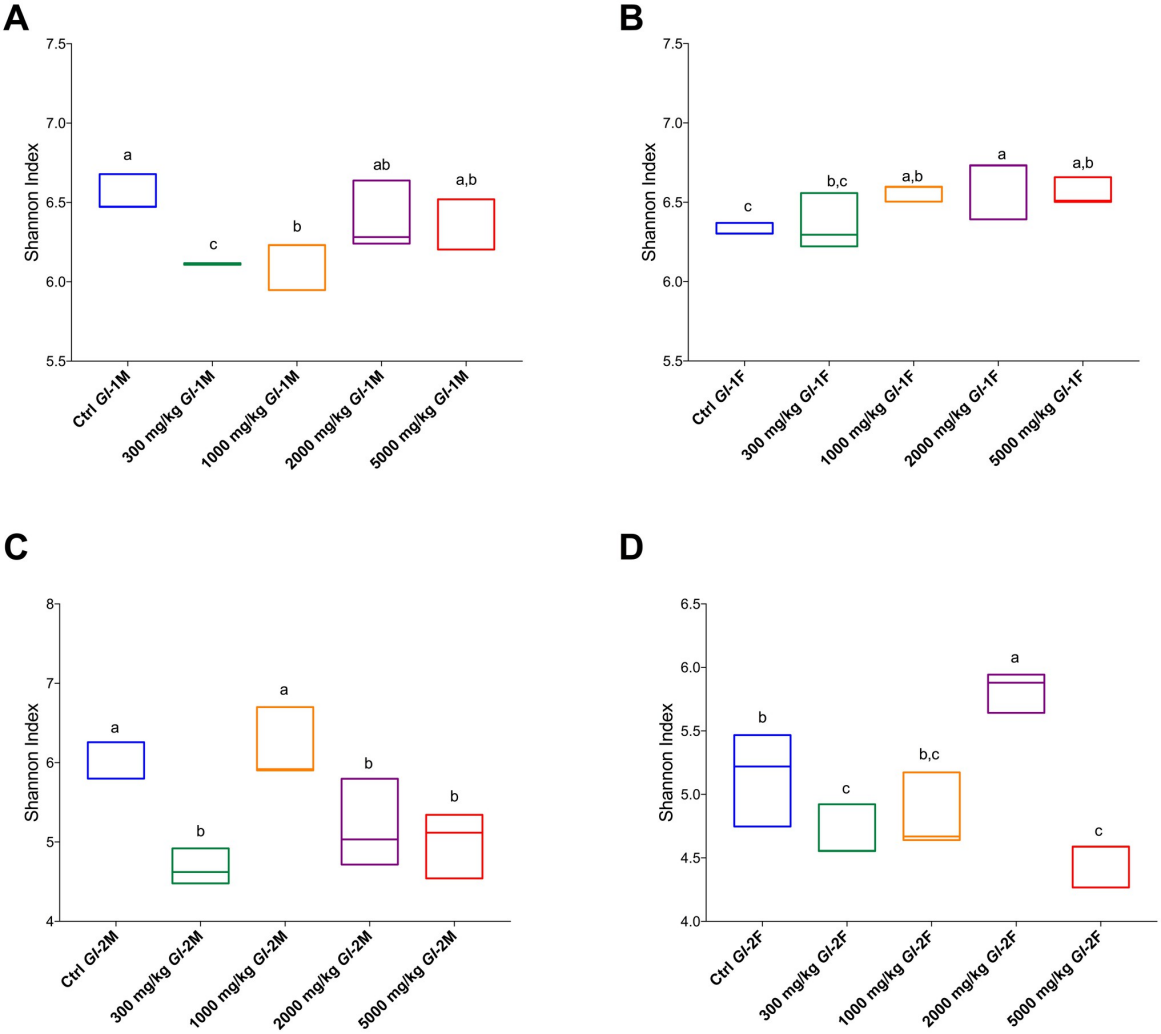
Comparative alpha diversity in the gut microbiota from the male (M) or female (F) Wistar rats fed with the control diet (Ctrl) and different doses of standardized *Gl*-1 or *Gl*-2 extracts of *Ganoderma lucidum* for 14 experimental days, according to the Shannon index. A–B: Administration of the *Gl*-1 extract. C–D: Administration of the *Gl*-2 extract. The dose administered per experimental group is described in the materials and methods section. Different letters on a box indicate significant differences, *p* < 0.05. Values (*p*) correspond to one-way ANOVA from Tukey’s multiple comparison test.

**Fig 14:**
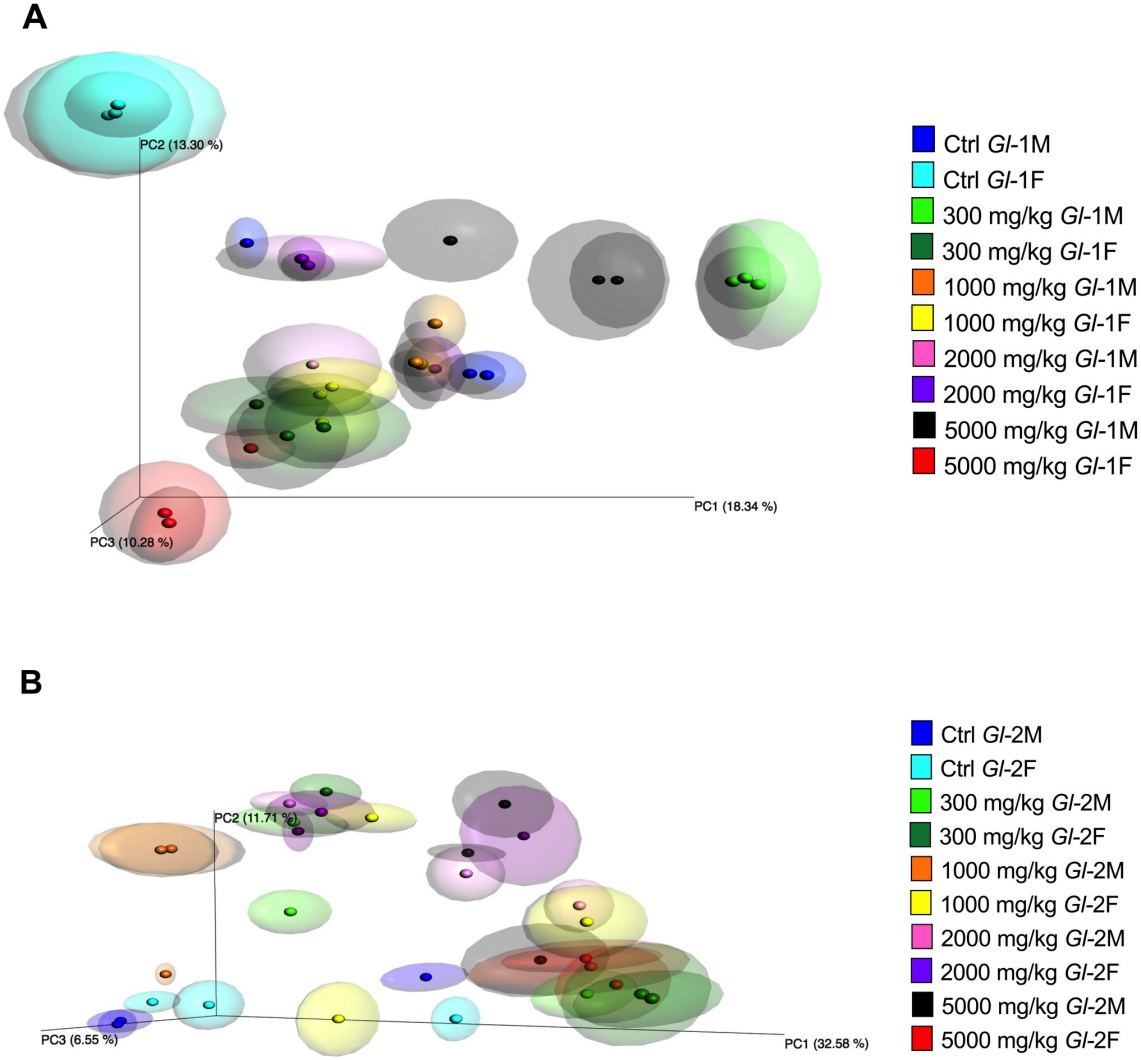
Principal coordinates analysis of beta diversity in the gut microbiota among male (M) or female (F) Wistar rats fed with the control diet (Ctrl) and different doses of standardized *Gl*-1 or *Gl*-2 extracts of *Ganoderma lucidum* for 14 experimental days. A: Administration of the *Gl*-1 extract. B: Administration of the *Gl*-2 extract. The dose administered per experimental group is described in the materials and methods section.

All animals consuming *Gl* extracts showed high diversity in the gut microbiota according to the Shannon index (>4). Alpha diversity in the gut microbiota within each experimental rat group studied is shown in Fig 13, as calculated by OTUs recorded and Chao 1 and Shannon indices. There were significant differences in species richness and diversity between the gut microbiota of each treated rat group and those from the control group. The only exception was a male group consuming the *Gl*-2 extract (1000 mg/kg dose). The comparison of beta diversity by principal coordinates analysis (PCoA) showed compositional differences in the gut microbiota among treated rat groups consuming different doses of *Gl*-1 or *Gl*-2 extracts. Although there is some overlap, most rats consuming the same dose, either *Gl*-1 or *Gl*-2 extract, were grouped together or close to each other, and independently from control groups (Fig 14).

**Fig 15:**
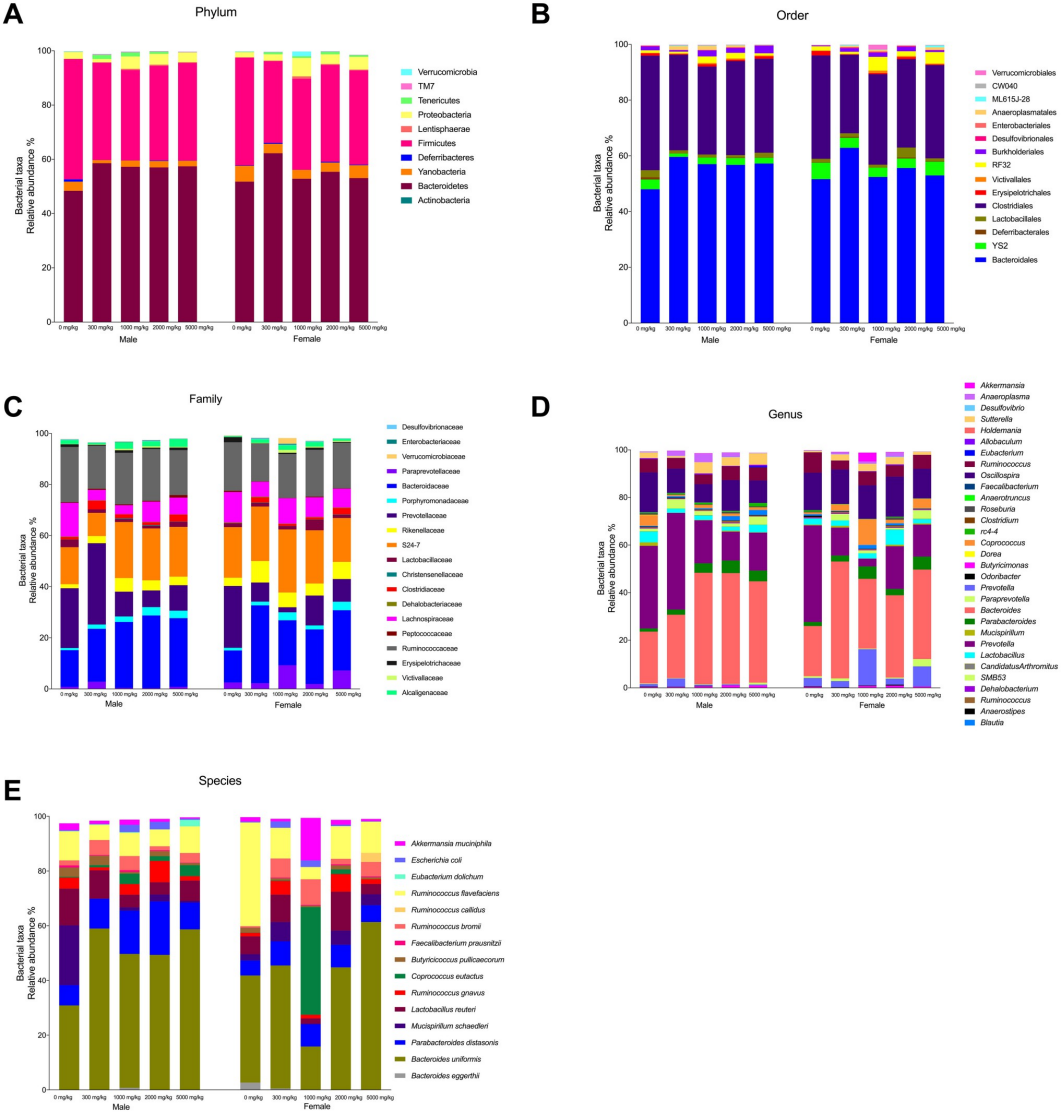
Effect of different doses of standardized *Gl*-1 extract of *Ganoderma lucidum* on the taxonomic distribution of bacterial communities from the gut microbiota of male (M) or female (F) Wistar rats for 14 experimental days. Main average bacterial relative abundance (BRA) is presented at the following taxonomic ranks: Phyllum (A), Order (B), Family (C), Genus (D), and Species (E). The dose administered per experimental group is described in the materials and methods section. Scientific names according to international database (http://www.catalogueoflife.org/).

At the phylum level, ten phyla were identified in rat groups fed with the *Gl*-1 extract. In comparison with the control group, consumption of the *Gl*-1 extract by male and female Wistar rats increased the bacterial relative abundance (BRA) of phyla Bacteroidetes (>19% males, >7.9% females), Proteobacteria (>33.4% males, >128.2% females), Tenericutes (>977.7% males, >380% females), and Lentisphaerae (>312.5% males, >633.3% females), whereas BRA decreased in the phyla Cyanobacteria (<42.3% males, <36.1% females) and Firmicutes (<21% males, <15.7% females) (Fig 15A). Different taxa were identified at order (19), family (28), and genus (35) levels (Fig 15B–15E). BRA greater than the control was recorded in *Parabacteroides*, *Bacteroides*, *SMB53*, *Prevotella*, and *Sutterella*. BRA decreased in *Ruminococcus*, and *Oscillospira* (Fig 15D). Differential effect of the *Gl*-1 extract on BRA was recorded in *Lactobacillus* (<43.5% males, >28.6% females), and *Akkermansia* (<30.0% males, >404.7% females), decreasing in male rats, whereas increasing in female rats. Twenty different bacterial species were identified in male and female rat groups consuming the *Gl*-1 extract (Fig 15E). Main species accounting for 95.9–97.0% of total BRA in rat groups fed with the *Gl*-1 extract were *Bacteroides uniformis* (55.2% males, 42.4% females), *Parabacteroides distasonis* (13.9% males, 7.1% females), *Ruminococcus flavefaciens* (6.8% males, 10.3% females), *Lactobacillus reuteri* (6.5% males, 7.8% females), *Ruminococcus bromii* (4.0% males, 5.7% females), *Ruminococcus gnavus* (3.5% males, 3.1% females), *Coprococcus eutactus* (2.1% males, 10.3% females), *Escherichia coli* (1.3% males, 1.2% females), *Akkermansia muciniphila* (1.1% males, 4.7% females), and *Mucispirillum schaedleri* (1.1% males, 3.9% females). BRA of main species increased in male and female rat groups consuming the *Gl*-1 extract, in comparison with the control group (Fig 15E), including *Bacteroides uniformis* (>73.3% males, >9.4% females), *Parabacteroides distasonis* (>94.7% males, >31.7% females), *Coprococcus eutactus* (>593.5% males, >12,787.5% females), *Ruminococcus bromii* (>146.0% males, >1,121.2% females), and *Escherichia coli* (>283.3% males, >281.8% females). The BRA of *Ruminococcus flavefaciens* decreased in all treated groups (<42.7% males, <72.4% females). Differential effect of the *Gl*-1 extract was recorded on several species, including *Mucispirillum schaedleri* (<94.2% males, >75.2% females), *Akkermansia muciniphila* (<50.4% males, >203.2% females), *Lactobacillus reuteri* (<56.3% males, >23.0% females), and *Ruminococcus gnavus* (<3.5% males, >138.6% females), in which BRA decreased in male rats. In contrast, it increased in female rats, compared to the control group.

**Fig 16:**
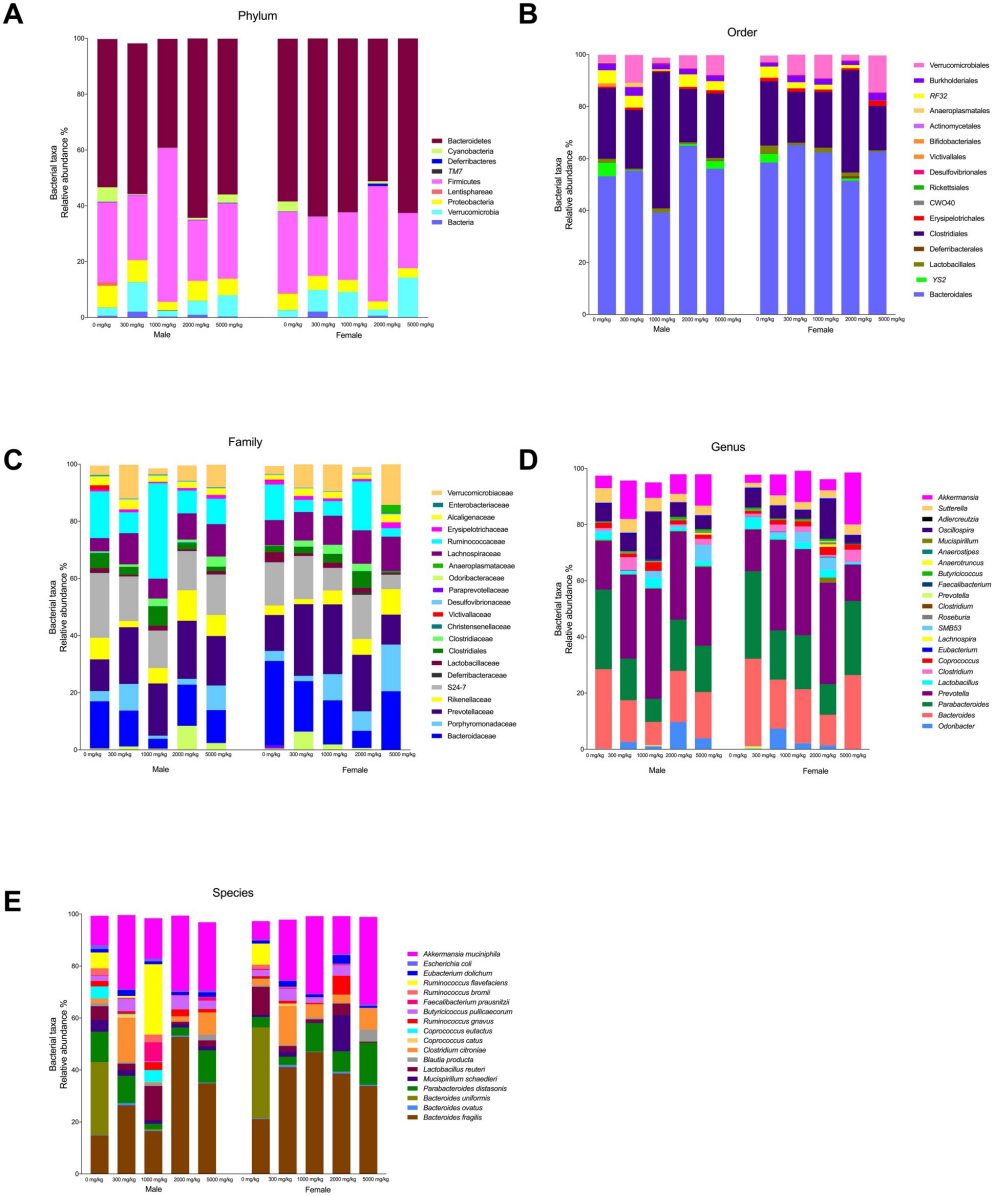
Effect of different doses of standardized *Gl*-2 extract of *Ganoderma lucidum* on the taxonomic distribution of bacterial communities from the gut microbiota of male (M) or female (F) Wistar rats after 14 experimental days. The main average bacterial relative abundance (BRA) is presented at the following taxonomic ranks: Phyllum (A), Order (B), Family (C), Genus (D), and Species (E). The dose administered per experimental group is described in the materials and methods section. Scientific names according to international database (http://www.catalogueoflife.org/).

Ten phyla were also identified in male and female Wistar rats consuming the *Gl*-2 extract and the control group (Fig 16A). In comparison with the control group, consumption of the *Gl*-2 extract by male and female Wistar rats increased BRA of phyla Bacteroidetes (>1.2% males, >2.7% females), and Verrucomicrobia (>74.2% males, >221.6% females), while the BRA of Cyanobacteria (<74.7% males, <93.6% females), Proteobacteria (<21.3% males, <33.4% females), and Firmicutes [<12.9% males (except 1000 mg/kg dose), <8.5% females] decreased (Fig 16A). There were various taxonomic ranks identified at order (20), family (30), and genus (36) levels (Fig 16B–16E). Genera *Prevotella*, *Akkermansia*, *Oscillospira*, *Odoribacter*, *SMB53*, and *Clostridium* showed BRA greater than the control in male and female Wistar rats consuming the *Gl*-2 extract, while *Parabacteroides*, *Bacteroides*, and *Lactobacillus* decreased. The *Gl*-2 extract had a differential effect on BRA recorded in *Sutterella* (<24.4% males, >69.4% females), and *Coprococcus* (<2.6% males, >103.8% females), decreasing in male rats, while increasing in female rats (Fig 16D). This was also the case for the BRA of several species (Fig 16E), including *Mucispirillum schaedleri* (<64.0% males, >368.3% females), *Blautia producta* (<13.0% males, >219.6% females), *Parabacteroides distasonis* (<40.0% males, >135.9% females), and *Ruminococcus gnavus* (<4.7% males, >117.2% females). Conversely, BRA of *Ruminococcus flavefaciens* (>14.6% males, <99.6% females), and *Faecalibacterium prausnitzii* (>985.7% males, <38.4% females) increased in male rats, but decreased in female rats. Twenty-five bacterial species were identified in male and female rat groups consuming the *Gl*-2 extract (Fig 16E). The main species accounting for 96.1–97.3% of total BRA in rat groups fed with the *Gl*-2 extract were *Bacteroides fragilis* (32.5% males, 40.0% females), *Akkermansia muciniphila* (24.8% males, 25.3% females), *Clostridium citroniae* (6.9% males, 7.8% females), *Parabacteroides distasonis* (6.9% males, 9.5% females), *Ruminococcus flavefaciens* (6.9% males, 0.03% females), *Lactobacillus reuteri* (4.6% males, 2.0% females), *Butyricicoccus pullicaecorum* (3.3% males, 2.7% females), *Faecalibacterium prausnitzii* (2.2% males, 0.2% females), *Ruminococcus gnavus* (1.9% males, 2.1% females), *Mucispirillum schaedleri* (1.6% males, 4.0% females), *Eubacterium dolichum* (1.5% males, 1.8% females), *Coprococcus eutactus* (1.2% males, 0.01% females), and *Blautia producta* (1.0% males, 1.4% females). BRA of several species increased in male and female rat groups consuming the *Gl*-2 extract compared to the control group (Fig 16E). These species were *Bacteroides fragilis* (>121.1% males, >90.2% females), *Akkermansia muciniphila* (>119.2% males, >256.1% females), *Clostridium citroniae* (>288.3% males, >213.9% females), *Butyricicoccus pullicaecorum* (>66.8% males, >12.5% females), and *Eubacterium dolichum* (>23.4% males, >55.0% females). By contrast, BRA of *Lactobacillus reuteri* (<11.0% males, <81.3% females), and *Coprococcus eutactus* (<73.6% males, <92.8% females) decreased in all treated groups, as compared to the control group.

## Discussion

Extensive potential therapeutic applications of functional and medicinal properties of *Ganoderma lucidum*, derived from their traditional use and current commercial production, are the initial backing for the safety of a variety of products and bioactive compounds. They include drugs, herbal remedies, extracts, dietary supplements, health foods, beverages, powdered basidiocarps, spore powders, and cosmetics, involving various dosage forms by ingestion (tea, decoction, food, bolus, syrup, tablet, capsule, tincture), injection (solution), or spreading (cream, ointment). VanderMolen et al. [46] detected a market presence of 315 products in the USA. Although only very few products and dosages are supported by scientific evidences and thorough toxicity studies, the safety of *G*. *lucidum* by oral administration has been confirmed using *in vivo* models, under various experimental conditions [10, 14–22].

Our results showed that hydroalcoholic extracts from a Mexican genotype of *G*. *lucidum*, cultivated on oak sawdust (*Gl*-1) or oak sawdust plus ASA (*Gl*-2), had no relevant adverse effects or toxicity on male and female Wistar rats studied. Animals were active and healthy at the end of the study in all tested doses administered orally, as compared to control groups.

There was a dose-related association found in male and female rat groups consuming the highest doses of *Gl*-1 or *Gl*-2 extracts (5000 mg/kg), being temporarily more active than the other groups, which has not previously been reported. The presence of low concentrations of compound(s) in *Gl* extracts responsible for normal active behavior is possible, suggesting a stimulatory effect analogous to caffeine in small amounts [47]. There were no other behavioral changes, external clinical signs, alterations, allergies, or mortalities in all treated (*Gl*-1 or *Gl*-2 extracts) or untreated (controls) rat groups during this study.

Different doses studied of *Gl*-1 or *Gl*-2 extracts did not significantly affect food intake, weight gain, or organ weight (liver, kidney) of male and female Wistar rats, as well as important serum biochemical parameters (glucose, CRP, urea). However, there was a differential effect on several parameters depending on the type of extract and sex, which were not dose-dependent.

Serum and urinary levels of creatinine or urea are important biomarkers of renal function. The *Gl*-1 extract significantly reduced serum creatinine levels in treated male and female rats. There were no significant variations in serum creatinine levels of male and female rats consuming the *Gl*-2 extract. The excretion of urinary creatinine varied in male rats consuming *Gl*-1 or *Gl*-2 extracts (16.45–51.38 mg/dL); however, female rats showed greater variations (12.47–47.43 mg/dL). Serum urea varied in male and female rats consuming *Gl*-1 or *Gl*-2 extracts (males: 4.37–6.48 mmol/L; females: 3.29–6.76 mmol/L). The excretion of urinary urea nitrogen also showed variation in treated male (555.20–1,483.0 mg/24 h) and female (448.50–1,248.0 mg/24 h) groups consuming *Gl*-1 or *Gl*-2 extracts.

Another biomarker of kidney function is urinary albumin. Male and female rats consuming *Gl*-1 or *Gl*-2 extracts showed variations of urinary albumin (males: 0.29–5.30 mg/dL; females: 0.16–2.73 mg/dL). *Gl*-1 and *Gl*-2 extracts showed a differential effect on the albumin-to-creatinine ratio (ACR). In male and female rats, the *Gl*-1 extract led to a moderate decrease or increase of the ACR (8.0–279.0 mg/g), whereas the *Gl*-2 extract to a mildly decrease or increase of the ACR (4.9–12.5 mg/g).

Serum glucose varied in male and female rats consuming *Gl*-1 or *Gl*-2 extracts (males: 150.0–171.40 mg/dL; females: 149.40–165.70 mg/dL). In the urine test, glucose levels also varied in male and female rats consuming *Gl*-1 or *Gl*-2 extracts (males: 3.93–10.0 mg/24 h; females: 3.33–7.50 mg/24 h).

A comparison between urinary biochemical parameters recorded in control male and female Wistar rats and those from previous research showed differences. Although these parameters have been poorly studied, Bazzano et al. [48] reported lower or higher values for glucose (males: 5.25±1.25 mg/dL; females: 2.75±0.48 mg/dL), urea (males: 1,753.23±343.41 mg/dL; females: 1,096.24±142.55 mg/dL), and creatinine (males: 37.55±5.72 mg/dL; females: 40.59±7.64 mg/dL) in Wistar rats. In the case of albumin, Alter et al. [49] reported higher data (21.6±16.8 mg/24 h) in male Wistar rats. Comparative analyses are difficult due to these fluctuations, which can be attributed not only to experimental diets, but also to animal characteristics (genotype, sex, age, weight), analytical methods, experimental conditions, and the lack of protocol standardization.

At the molecular level, important injury and inflammation biomarkers were studied in kidney tissue, particularly KIM-1/TIM-1, TLR4, and NF-кB proteins. There was a differential effect of *Gl*-1 and *Gl*-2 extracts on the concentration of KIM-1/TIM-1 protein determined in kidney tissue. Male rat groups consuming *Gl*-1 or *Gl*-2 extracts showed significantly lower concentrations of the KIM-1/TIM-1 protein than control groups. However, no significant effect was recorded in female rats consuming the *Gl*-1 extract, whereas most groups consuming the *Gl*-2 extract had significantly lower concentrations of the KIM-1/TIM-1 protein than the control group. Expressions of TLR4 and NF-кB proteins were not affected by *Gl*-1 or *Gl*-2 extracts consumed by male and female rats, showing that no significant immune and/or inflammation responses were activated in the kidney.

Data analysis indicated that, in general, there were no significant abnormal variations in organ weight, serum biochemical parameters (CRP, creatinine, urea, glucose), urinary parameters (creatinine, urea nitrogen, albumin, ACR, glucose), injury and inflammation biomarkers (KIM-1/TIM-1, TLR4, NF-кB proteins), showing no kidney injury or renal dysfunction derived from the consumption of *Gl*-1 or *Gl*-2 extracts by male and female Wistar rats receiving doses of 300 mg/kg, 1000 mg/kg, 2000 mg/kg, and 5000 mg/kg. Histopathological examination of kidneys from male and female rat groups consuming *Gl*-1 or *Gl*-2 extracts confirmed this finding, showing no abnormal morphological changes in renal glomeruli, Bowmanʼs capsules, or tubules, in comparison with control groups. However, potential toxicity targets were identified in a few rat groups (5–20%), in which higher values not correlated with other parameters were recorded in urinary glucose, urea nitrogen, creatinine, and albumin.

Several parameters also assessed liver function. There was a general trend towards a significant reduction of serum ALT and AST transaminases in most male and female rat groups consuming *Gl*-1 or *Gl*-2 extracts. In male rats, the ALT concentration varied from 39.80–76.02 U/L, while the AST concentration ranged from 139.30–243.10 U/L. In female rats, ALT and AST concentrations were 35.70–72.47 U/L and 153.10–305.50 U/L, respectively. Further analysis at the molecular level showed no inflammatory responses in the liver after administration of *Gl*-1 or *Gl*-2 extracts. There were no significant differences in the liver expression of the IL-1β protein between male and female rat groups treated with the *Gl*-2 extract, and their control groups. By contrast, the *Gl*-1 extract induced a significant reduction in the liver expression of the IL-1β protein in male and female rat groups, as compared to their control groups. This differential effect showed that the *Gl*-1 extract affects the expression of the IL-1β protein in the liver. This was also the case for female rat groups, in which the expression of TNF-α proinflammatory mediator was also significantly reduced by the *Gl*-1 extract. However, in male rat groups, the expression of the TNF-α protein was significantly reduced by *Gl*-1 and *Gl*-2 extracts in two groups only (300 mg/kg, 5000 mg/kg). In comparison, the other five groups were not significantly different. Analysis of the IL-6 proinflammatory mediator showed no significant differences between male and female rat groups consuming the *Gl*-1 extract and their control groups. This was so for female rat groups consuming the *Gl*-2 extract and their control group. In the case of male rat groups consuming the *Gl*-2 extract, two groups were showing greater expression of the IL-6 protein at high doses (2000 mg/kg, 5000 mg/kg), and one group (1000 mg/kg) showing IL-6 protein expression significantly reduced, as compared to the control group. However, this greater expression of the IL-6 protein was atypical and not associated to: 1) Abnormal organ weight; 2) Abnormally high expression of other biomarkers studied (IL-1β, TNF-α); 3) Greater concentration of serum levels of ALT and AST transaminases; and 4) Abnormal morphology in the liver after histopathological examination.

The expression of other genes associated to cholesterol metabolism in the liver was also studied. Most male and female rat groups consuming *Gl*-1 or *Gl*-2 extracts showed equivalent or lesser expression of HMG-CoA reductase, Srebp2, and Ldlr genes compared to those of the control groups. There were only five exceptions recorded in the Ldlr gene, one following the administration of the *Gl*-1 extract and the others following the administration of the *Gl*-2 extract. The female rat group receiving the greatest dose of the *Gl*-1 extract (5000 mg/kg) showed a significantly higher expression of the Ldlr gene than the control group. In the case of female rat groups consuming the *Gl*-2 extract, expression of the Ldlr gene was significantly higher in three different doses (300 mg/kg, 1000 mg/kg, 5000 mg/kg). Greater expression of the Ldlr gene was only recorded in a male rat group fed with the lowest dose (300 mg/kg) of the *Gl*-2 extract. Another similar exception was a greater expression of the Srebp2 gene, only recorded in the male rat group consuming the lowest dose (300 mg/kg) of the *Gl*-2 extract. These atypical expressions of the Ldlr and Srebp2 genes were not associated to: 1) Abnormal organ weight; 2) Abnormally high expression of other biomarkers studied (IL-1β, TNF-α, IL-6); 3) Greater concentration of serum levels of ALT and AST transaminases; and 4) Abnormal morphology in the liver after histopathological examinations. This study confirmed that *Gl*-1 and *Gl*-2 extracts modulate down-regulation and up-regulation of important genes associated with cholesterol biosynthesis and the liver uptake of LDL-c (HMG-CoA reductase, Srebp2, Ldlr), as it has previously been reported by the authors [32].

Serum biochemical parameters showed no alterations or drastic effects following the administration of different doses of *Gl*-1 or *Gl*-2 extracts on cholesterol metabolism in the liver. Serum total cholesterol (TC) was not affected in most male rat groups consuming *Gl*-1 (51.05–59.58 mg/dL) or *Gl*-2 (51.0–52.63 mg/dL) extracts, in comparison with control groups (50.96–62.89 mg/dL). Only two male rat groups receiving high doses of the *Gl*-2 extract (2000 mg/kg, 5000 mg/kg) showed greater serum cholesterol (65.72–68.20 mg/dL) than the control group. Similar trend was also recorded in other parameters, LDL-c (*Gl*-1: 7.14–10.63 mg/dL, *Gl*-2: 8.41–10.08 mg/dL, controls: 8.34–9.60 mg/dL), and TG (*Gl*-1: 71.89–76.66 mg/dL, *Gl*-2: 68.21–98.59 mg/dL, controls: 64.37–86.80 mg/dL). Interesting was the case of the HDL-c parameter, which had a positive increase in all doses administered to male rat groups (*Gl*-1: 47.29–57.93 mg/dL, *Gl*-2: 43.76–65.14 mg/dL), as compared to control groups (36.98–48.58 mg/dL). In most female rat groups, TC was not affected or was reduced after the consumption of *Gl*-1 (46.72–54.26 mg/dL) or *Gl*-2 (59.07–69.38 mg/dL) extracts, in comparison with control groups (51.60–67.62 mg/dL). Only one female rat group receiving an intermediate dose of the *Gl*-2 extract (1000 mg/kg) showed greater serum cholesterol (76.66 mg/dL) than the control groups. Other parameters were also not affected or showed positive lowering trends, including LDL-c (*Gl*-1: 5.95–10.05 mg/dL, *Gl*-2: 11.11–12.43 mg/dL, controls: 10.20–11.80 mg/dL), and TG (*Gl*-1: 70.96–81.42 mg/dL, *Gl*-2: 52.98–69.98 mg/dL, controls: 54.79–74.86 mg/dL). The HDL-c parameter showed a positive increase in most female rat groups consuming doses studied (*Gl*-1: 45.60–60.36 mg/dL, *Gl*-2: 54.67–61.80 mg/dL), as compared to control groups (48.10–57.23 mg/dL). The positive increase of HDL-c parameter in male and female rats is consistent with a previous report in exploratory clinical trials administering the *Gl*-1 extract [2].

Data analysis showed, in general, no liver injury or hepatic dysfunction after the consumption of *Gl*-1 or *Gl*-2 extracts by male and female Wistar rats receiving doses of 300 mg/kg, 1000 mg/kg, 2000 mg/kg, and 5000 mg/kg, as there were no significant abnormal variations in organ weight, serum biochemical parameters (ALT and AST transaminases, TC, LDL-c, TG, HDL-c), inflammation biomarkers (IL-1β, TNF-α, and IL-6 proteins), or the expression of genes linked to cholesterol metabolism (HMG-CoA, Srebp2, Ldlr). Histopathological analysis of livers from male and female rat groups consuming *Gl*-1 or *Gl*-2 extracts confirmed this result, showing the structural integrity of hepatocyte cell and cell membranes, no morphological abnormalities, and no hepatic cells swelling. However, potential targets of toxicity were identified in a few rat groups (10–20%), in which higher values not correlated with other parameters were recorded in AST transaminase (300 mg/kg *Gl*-1F; 5000 mg/kg *Gl*-2F), LDL-c (2000, 5000 mg/kg *Gl*-1M), TG (2000, 5000 mg/kg *Gl*-1F), and cholesterol (2000, 5000 mg/kg *Gl*-2M; 300, 1000 mg/kg *Gl*-2F).

Analysis of the gut microbiota in stool samples confirmed the prebiotic effects of *Gl*-1 and *Gl*-2 extracts, as previously reported by the authors [32]. Analyses of alpha and beta diversity also confirmed the significant differential effect of *Gl*-1 or *Gl*-2 extracts on the gut microbiota within each rat group in terms of species richness, divergence or evenness, as well as on compositional differences between experimental rat groups. The results provided further evidence of the differential modulation of each extract (*Gl*-1, *Gl*-2) on several bacterial taxa, as well as their effects on male and female Wistar rats. These effects showed no dose-dependent patterns. The bacterial diversity increased 2.9–25% and 2.9–19.0% in most taxonomic ranks of the gut microbiota from rat groups consuming the *Gl*-2 extract, in comparison with the *Gl*-1 extract (Order: >5.3%; Family: >7.1%; Genus: >2.9%; Species: >25.0%) and controls (Order: >5.3%; Family: >3.4%; Genus: >2.9%; Species: >19.0%), respectively. In general, as compared to the controls, both *Gl*-1 and *Gl*-2 extracts promoted increased BRA of obligate anaerobic bacteria belonging to phylum Bacteroidetes (*Gl*-1: >19% males, >7.9% females; *Gl*-2: >1.2% males, >2.7% females), while the abundance of phyla Firmicutes decreased (*Gl*-1: <21.0% males, <15.7% females; *Gl*-2: <12.9% males, <8.5% females). Thus, the Firmicutes/Bacteroidetes ratio is positively modulated, as the opposite BRA ratio (*i*.*e*., increased Firmicutes and reduced Bacteroidetes), has been correlated with a less beneficial metabolic profile, and associated to obese individuals [50]. The consumption of *Gl*-1 or *Gl*-2 extracts by rat groups decreased the BRA of phylum Cyanobacteria, whereas genera *Prevotella* and *SMB53* increased. There was a clear differential effect on the phylum Proteobacteria, which increased in rat groups consuming the *Gl*-1 extract, but decreased in those groups consuming the *Gl*-2 extract. This was also the case for genera *Parabacteroides* and *Bacteroides*, as well as for *Coprococcus eutactus*. The opposite effect was recorded in *Oscillospira*, as BRA decreased in rat groups consuming the *Gl*-1 extract, but increased in those groups consuming the *Gl*-2 extract. There were several cases of differential effects between male and female rat groups consuming *Gl* extracts. *Mucispirillum schaedleri* and *Ruminococcus gnavus* decreased in all male rat groups treated with *Gl*-1 or *Gl*-2 extracts, while they increased in all female rat groups. This differential effect was also recorded in other bacterial genera and species for *Gl*-1 or *Gl*-2 extracts. The *Gl*-1 extract exerted this effect on *Lactobacillus reuteri* and *Akkermansia muciniphila*, whereas the *Gl*-2 extract did so on *Sutterella*, *Coprococcus*, *Blautia producta*, and *Parabacteroides distasonis*. Similar results of increased relative abundance of *Sutterella* were reported by Guo et al. [51], using an ethanol extract of *Gl*. This genus has been associated with inflammatory bowel disease and metabolic syndrome [52]. The opposite effect was also observed, the *Gl*-2 extract increased BRA of *Ruminococcus flavefaciens* and *Faecalibacterium prausnitzii* in male rats, while it decreased BRA of these species in female rats. The BRA also increased in other taxa depending on the extract, including Tenericutes (*Gl*-1), Lentisphaerae (*Gl*-1), Verrucomicrobia (*Gl*-2), *Odoribacter* (*Gl*-2), *Bacteroides uniformis* (*Gl*-1, *Gl*-2), *Parabacteroides distasonis* (*Gl*-1), *Ruminococcus bromii* (*Gl*-1), *Escherichia coli* (*Gl*-1), *Akkermansia muciniphila* (*Gl*-2), *Clostridium citroniae* (*Gl*-2), *Butyricicoccus pullicaecorum* (*Gl*-2), and *Eubacterium dolichum* (*Gl*-2). By contrast, the BRA decreased in *Ruminococcus flavefaciens* (*Gl*-1) and *Lactobacillus reuteri* (*Gl*-2). *Ruminococcus* has been associated with irritable bowel syndrome and pro-inflammatory responses [50].

Gut microbiota analysis after the consumption of *Gl*-1 or *Gl*-2 extracts by Wistar rat groups, in comparison with their controls, showed that: 1) Bacterial diversity and BRA increased in different taxa; 3) The Firmicutes/Bacteroidetes ratio was positively modulated; 4) Different specific taxa, including phyla, genera and species, were shown to be sensitive to either *Gl*-1 or *Gl*-2 extracts, or both, leading to increased or decreased BRA in the gut microbiota; 5) There was a differential effect of *Gl* extracts on BRA of several species being sex dependent; and 6) There were no signs of adverse effects or gastrointestinal disturbances (pain, diarrhea, vomiting) derived from changes in the gut microbiota. Similar results on gut microbiota regulation have been reported in other mushroom species [53].

Overall results showed no significant adverse, toxic, or harmful effects after oral administration of different high doses of standardized *Gl*-1 or *Gl*-2 extracts to male and female Wistar rats for 14 days, compared to control groups. Several parameters fluctuated slightly and irregularly as part of the normal biological variation or reaction. They were not associated to the dose-response relationship, so they were considered without toxicological significance in the risk/benefit analysis. Some parameters were associated with the beneficial effects of *G*. *lucidum* previously reported (hypocholesterolemic, anti-inflammatory, and prebiotic properties). A few parameters were identified as potential targets of toxicity to be followed in further longer-term toxicity studies (urinary glucose, urea nitrogen, creatinine, albumin; serum AST transaminase, LDL-c, TG, and cholesterol), mainly in male and female rats treated with higher doses of *Gl*-1 or *Gl*-2 extract (2000, 5000 mg/kg dose). These findings are consistent with previous studies on the acute, subchronic, and genetic toxicology of diverse compounds (β-glucans, polysaccharides, polysaccharide peptide, triterpenes) and products (extracts from mycelium, basidiocarps, or spores) from *G*. *lucidum* [17–23]. We found that the ASA (10 mM) added to the substrate used for mushroom cultivation, not only changed the chemical composition of the *Gl*-2 extract, in comparison with the control (*Gl*-1, no ASA), but also its properties and effects on an *in vivo* model after administering high doses [2, 32]. There were clear differences between *Gl*-1 and *Gl*-2 extracts in most parameters assessed in treated Wistar rats. Further differences between these *Gl* extracts have also been shown recently by the authors at the genomic level [34]. The no-observed-adverse-effect-level (NOAEL) [54] was 1000 mg/kg bw/day of *Gl*-1 or *Gl*-2 extracts, as this dose did not cause toxicologically relevant adverse effects in terms of frequency or severity. The tolerance of treated Wistar rats to this high dose is remarkable. In an oral subchronic study, Chen et al. determined a NOAEL of 2000 mg/kg bw/day for a high purity standardized β-glucan extracted from *G*. *lucidum*, the highest dose tested in male and female Sprague Dawley rats after 13 weeks [17]. Smina et al. showed no toxic effect at 5000 mg/kg bw dose of total triterpenes of *G*. *lucidum* in an oral acute toxicity study for 14 days [18]. No significant effects on hematology, kidney or liver function at a high dose of 1200 mg/kg/day were reported by Wihastuti et al. in Wistar rats fed with a polysaccharide peptide from *G*. *lucidum* in a subchronic toxicity study for 90 days [19–21]. Other studies showed no adverse effects or toxicity at a dose of 7.0 g/kg/day for a chitin-glucan from *Aspergillus niger* administered orally to Wistar rats for 13 weeks, and 5.9 g/kg/day for a barley β-glucan given orally to Wistar rats for 28 days [55, 56]. *Gl*-1 and *Gl*-2 extracts altered the composition of gastrointestinal bacteria in Wistar rats, suggesting that the gut microbiota was a major biological mechanism for achieving homeostasis in treated groups. Bacterial diversity (>2.9–25%) and relative bacterial abundance (>1.2–19%) increased in most taxonomic ranks after treatment, showing that *Gl* extracts are absorbed by the gut microbiota and contributed to maintaining the metabolic activity of rats without toxicologically relevant or severe effects. This biological mechanism is variable as it depended on the gut microbiota composition in each rat, which explains why several parameters studied showed fluctuating or no dose-dependent patterns. Data from this study, as well as others from the authors, allow to determine the margin of safety (MOS = NOAEL/effective dose) [54], being as high as 3,496.5 for the *Gl* extracts, considering an effective dose of 0.286 mg/kg bw/day reported in exploratory clinical trials [2]. The overall risk/benefit analysis indicated that *Gl*-1 and *Gl*-2 extracts are, therefore, safe and unlikely to be harmful to human health administering the effective dose. The safety of *G*. *lucidum* is confirmed, as previous toxicity analyses have been followed by mutagenicity and DNA damaging studies, pharmacokinetics, as well as clinical trials [16, 17, 26–31, 57–60]. Further chronic oral toxicity and bioavailability studies can be carried out considering longer duration, reversibility, and different doses of *Gl* extracts for assessing their efficacy, the essential role of the gut microbiota (bacterial composition, quantity, activity, metabolites produced), and even potential precursors of serious effects or pharmacodynamic responses.

## Conclusions

Overall results of this repeated dose oral toxicity study showed that no significant adverse, toxic, or harmful effects were recorded on male or female Wistar rats after the oral administration of different doses (300 mg/kg, 1000 mg/kg, 2000 mg/kg, 5000 mg/kg) of well-characterized and standardized *Gl*-1 or *Gl*-2 extracts from a Mexican genotype of *G*. *lucidum*. Although several parameters fluctuated between treated and control groups, comparative biological and statistical analyses of variables studied revealed no toxicologically relevant effects. Analyses of kidney and liver revealed no injury or dysfunctions, as there were no significant abnormal variations of organ weight, tissue histopathology, serum biochemical parameters (CRP, creatinine, urea, glucose, ALT and AST transaminases, TC, LDL-c, TG, HDL-c), urinary parameters (creatinine, urea nitrogen, albumin, ACR, glucose), injury and inflammation biomarkers (KIM-1/TIM-1, TLR4, NF-кB proteins; IL-1β, TNF-α, IL-6 genes), or the expression of genes linked to cholesterol metabolism (HMG-CoA, Srebp2, Ldlr). *Gl*-1 and *Gl*-2 extracts showed prebiotic effects on the gut microbiota of male and female Wistar rats. However, alpha and beta diversity analyses revealed a significant differential effect within each rat group (species richness, divergence, evenness) and compositional differences between experimental rat groups. Bacterial diversity and relative bacterial abundance (BRA) increased in most taxonomic ranks after treatment, modulating positively the Firmicutes/Bacteroidetes ratio. Several specific taxa were sensitive to either *Gl*-1 or *Gl*-2 extracts or both. *Gl* extracts had a differential effect on the BRA of several species associated with the sex of rats. This evidence suggests that the gut microbiota was a primary biological mechanism for achieving homeostasis and maintaining the metabolic activity of treated rats without inducing toxicologically relevant effects. The acetylsalicylic acid (ASA, 10 mM) added to the substrate used for mushroom cultivation changed the properties and effects of the *Gl*-2 extract on the *in vivo* model, compared with the *Gl*-1 extract (no ASA). Observations in a few groups consuming higher doses (2000, 5000 mg/kg) of *Gl*-1 or *Gl*-2 extracts identified several parameters as potential targets of toxicity to be followed in future longer-term studies (urinary glucose, urea nitrogen, creatinine, albumin, AST transaminase, LDL-c, TG, cholesterol). The no-observed-adverse-effect-level (NOAEL) was 1000 mg/kg bw/day of *Gl*-1 or *Gl*-2 extracts, as this dose did not cause toxicologically relevant adverse effects, frequency or severity. Further studies and clinical trials can now be carried out for exploring potential therapeutic applications of *Gl*-1 and *Gl*-2 extracts, such as treatments for hypercholesterolemia, inflammation, modulation of the gut microbiota, oxidative stress, bacterial infections, as well as cancer prevention and inhibition.

## Supporting information

S1 Table Biochemical characterization of *Gl* extracts obtained from mature basidiomata of Mexican *Ganoderma lucidum*, cultivated on *Quercus* sawdust (*Gl*-1) substrate or *Quercus* sawdust substrate plus 10 mM acetylsalicylic acid (*Gl*-2). (XLSX)

S2 Table Composition of tested experimental diets and doses administered to male (M) and female (F) Wistar rats in this study, according to the standard AIN-93 diet. *Gl*-1: Extract from *Ganoderma lucidum* cultivated on the control substrate. *Gl*-2: Extract from *G*. *lucidum* cultivated on the treated substrate (ASA, 10 mM).
(DOCX)

S3 Table Primer sequences used to determine gene expression by reverse transcription polymerase chain reaction (RT-PCR). (DOCX)

S1 Raw images (PDF)
