# Supplementary material for: Effects of Mexican Ganoderma lucidum extracts on liver, kidney, and the gut microbiota of Wistar rats: A repeated dose oral toxicity study
Source: PLoS One. 2023 Apr 6;18(4):e0283605. doi: 10.1371/journal.pone.0283605 (PMC10079091; doi:10.1371/journal.pone.0283605)
Supplement: S2 Table — Gl-1: Extract from Ganoderma lucidum cultivated on the control substrate. Gl-2: Extract from G. lucidum cultivated on the treated substrate (ASA, 10 mM). (DOCX) [file pone.0283605.s002.docx]

**Supplementary Table 2.** Composition of tested experimental diets and doses administered to male (M) and female (F) Wistar rats in this study, according to the standard AIN-93 diet. *Gl*-1: Extract from *Ganoderma lucidum* cultivated on the control substrate. *Gl*-2: Extract from *G. lucidum* cultivated on the treated substrate (ASA, 10 mM).

| **Ingredients** | **Diet** |  | **Dose of *Gl* extract** | | | |
| --- | --- | --- | --- | --- | --- | --- |
|  | **Ctrl**  **(g/kg)** |  | **300 mg/kg** | **1000 mg/kg** | **2000 mg/kg** | **5000 mg/kg** |
| L-Cystine | 3.0 |  | 3.0 | 3.0 | 3.0 | 3.0 |
| Choline | 2.5 |  | 2.5 | 2.5 | 2.5 | 2.5 |
| Vitamins | 10.0 |  | 10.0 | 10.0 | 10.0 | 10.0 |
| Cellulose | 50.0 |  | 50.0 | 50.0 | 50.0 | 50.0 |
| Minerals | 35.0 |  | 35.0 | 35.0 | 35.0 | 35.0 |
| Soyabean oil | 70.0 |  | 70.0 | 70.0 | 70.0 | 70.0 |
| Starch | 397.5 |  | 397.5 | 397.5 | 397.5 | 397.5 |
| Dextrin | 132.0 |  | 132.0 | 132.0 | 132.0 | 132.0 |
| Saccharose | 100.0 |  | 100.0 | 100.0 | 100.0 | 100.0 |
| Casein | 200.0 |  | 200.0 | 200.0 | 200.0 | 200.0 |
| *Gl*-1 extract (*Gl*-1M, *Gl*-1F) | - |  | 300 | 1000 | 2000 | 5000 |
| *Gl*-2 extract (*Gl*-2M, *Gl*-2F) | - |  | 300 | 1000 | 2000 | 5000 |

*Gl*: *Ganoderma lucidum*. Ctrl: Control diet. ASA: Acetylsalicylic acid.
