# Supplementary material for: Effects of Mexican Ganoderma lucidum extracts on liver, kidney, and the gut microbiota of Wistar rats: A repeated dose oral toxicity study
Source: PLoS One. 2023 Apr 6;18(4):e0283605. doi: 10.1371/journal.pone.0283605 (PMC10079091; doi:10.1371/journal.pone.0283605)
Supplement: S3 Table — (DOCX) [file pone.0283605.s003.DOCX]

| **Gene** | **Sequence** | **Temperature**  **(^o^C)** |
| --- | --- | --- |
| S18 | Forward 5´-GGAGAGGGAGCCTGAGAA-AC | 64.2 |
|  | Reverse 5´-CAATTACAGGGCCTCGAA-AG |  |
| HMG-CoA | Forward 5´-GAGCTTGCTGTGAGAACG-TG | 64.2 |
|  | Reverse 5´-ACCTCCACCAAGACTGAT-CG |  |
| Ldlr | Forward 5´-ACCGCCATGAGGTACGTA-AG | 63.9 |
|  | Reverse 5`-CGGCGCTGTAGATCTTTC-TC |  |
| Srebp2 | Forward 5´-ACCTAGACCTCGCCAAAG-GT | 63.8 |
|  | Reverse 5´-CCTCGCACTGCTCTTAGC-TT |  |
| IL-6 | Forward 5´-ACCACCCACAACAGACCA-GT | 64.5 |
|  | Reverse 5´-CGGAACTCCAGAAGACCA-GA |  |
| TNF-α | Forward 5´-ATGTGGAACTGGCAGAGG-AG | 64.3 |
|  | Reverse 5´-GCCATGGAACTGATGAGA-GG |  |
| IL-1β | Forward 5´-CAGCAGCATCTCGACAAG-AG | 64.0 |
|  | Reverse 5´-CATCATCCCACGAGTCAC-AG |  |
|  |  |  |

**Supplementary Table 3.** Primer sequences used to determine gene expression by reverse transcription polymerase chain reaction (RT-PCR).

S18: Ribosomal protein 18. HMG-CoA: 3-hydroxy-3-methylglutaryl-CoA reductase. Ldlr: Low-density lipoprotein receptor. Srebp2: Sterol regulatory element-binding protein 2. IL-6: Interleukin 6. TNF-α: Tumor necrosis factor alpha. IL-1β: Interleukin 1 beta.
