# Supplementary figures and images for: Effects of Mexican Ganoderma lucidum extracts on liver, kidney, and the gut microbiota of Wistar rats: A repeated dose oral toxicity study
Source: PLoS One. 2023 Apr 6;18(4):e0283605. doi: 10.1371/journal.pone.0283605 (PMC10079091; doi:10.1371/journal.pone.0283605)

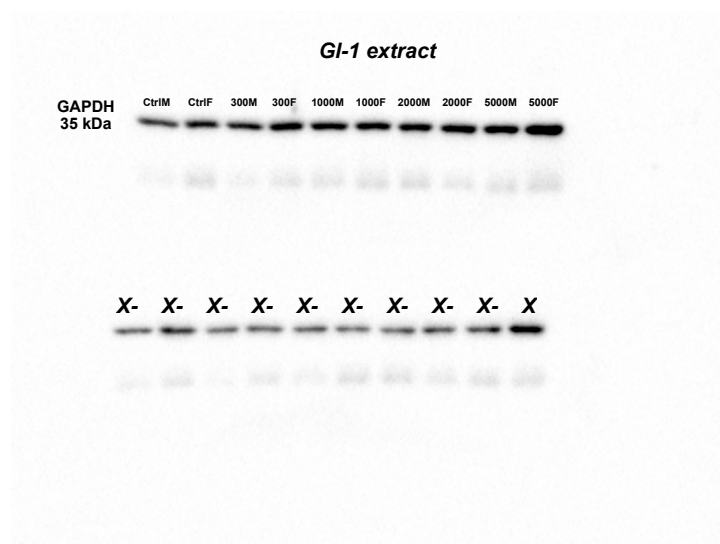

**FIG. 8**

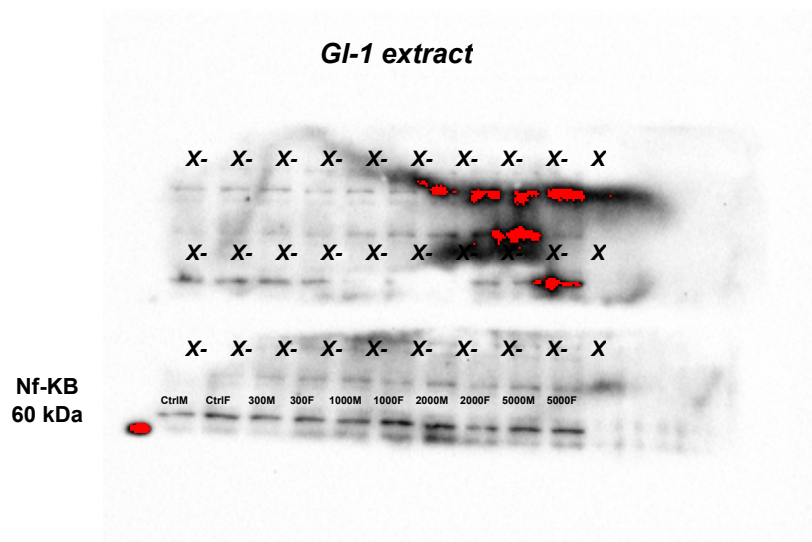

**FIG. 8**

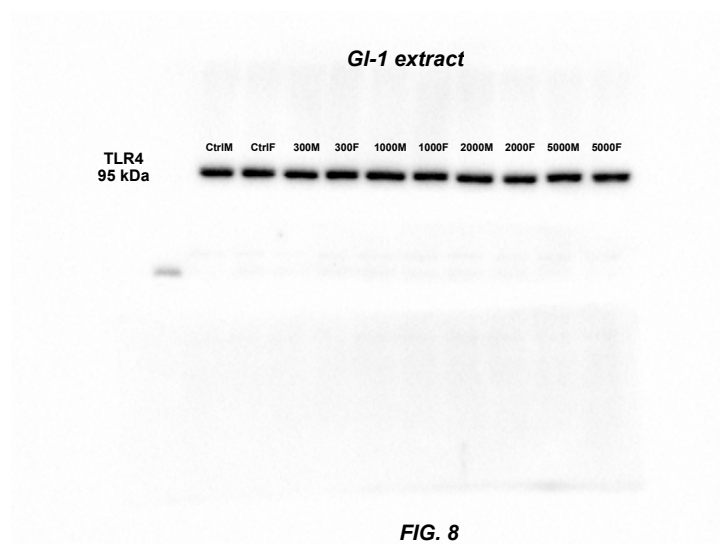

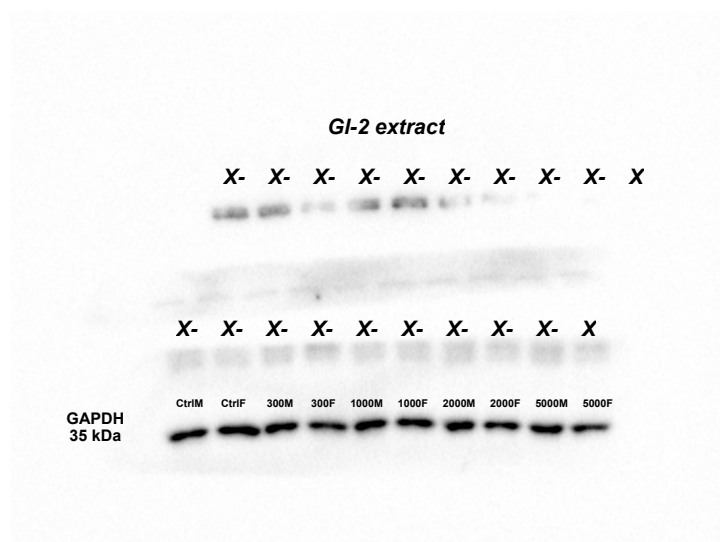

**FIG. 8**

**GI-2 extract**

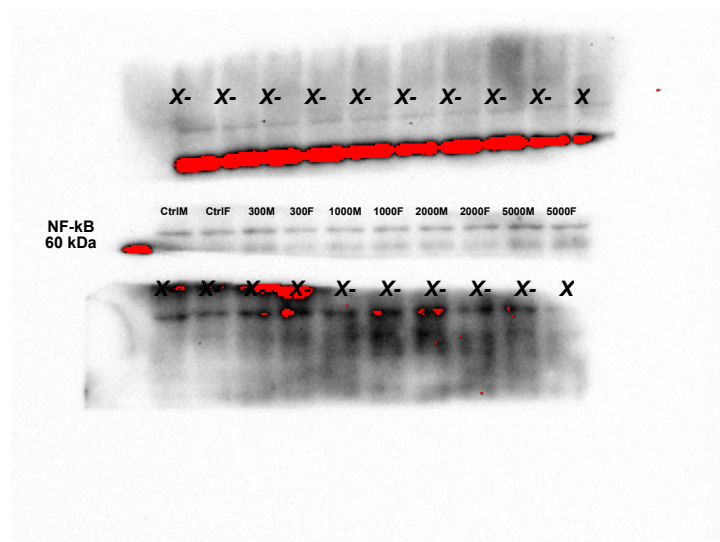

**FIG. 8**

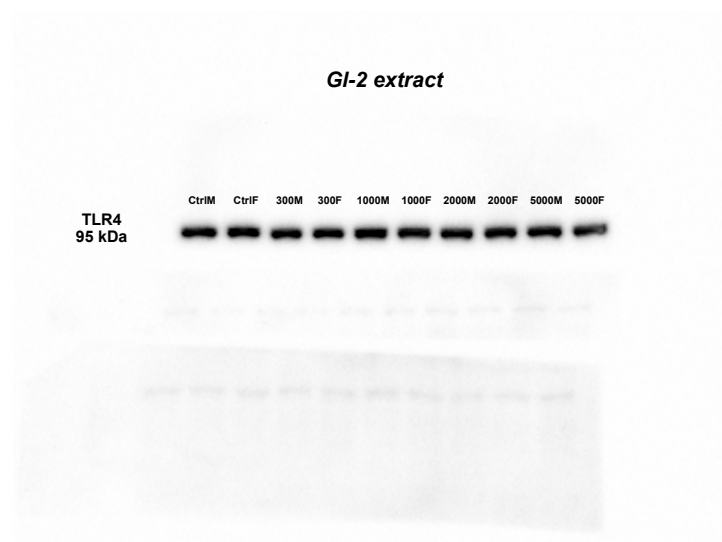

**FIG. 8**

Supplement: S1 Raw images — (PDF) [file pone.0283605.s004.pdf]
